# Continuous-flow ventilation with VENTIJET in moderate ARDS: a pilot safety and feasibility study

**DOI:** 10.1186/s40635-026-00876-7

**Published:** 2026-04-03

**Authors:** Lucía Picazo Moreno, Andrea Castellví-Font, Maria Acer Puig, Carles Camañes Mayordomo, Cristina Soriano Rodríguez, Joan Ramon Masclans Enviz, Francisco José Parrilla-Gómez

**Affiliations:** 1 Critical Care Department, Hospital del Mar de Barcelona. Critical Illness Research Group (GREPAC), Hospital del Mar Research Institute (HMRI), Passeig Marítim, 25-29, 08003 Barcelona, Spain; 2 Department of Medicine and Life Sciences (MELIS), Universitat Pompeu Fabra (UPF), Barcelona, Spain

**Keywords:** Acute respiratory distress syndrome (ARDS), Continuous-flow ventilation, Mechanical ventilation, VENTIJET

## Abstract

**Background:**

Acute respiratory distress syndrome (ARDS) remains associated with high mortality and ventilator-induced lung injury. Continuous-flow ventilation (CFV) may reduce cyclic strain, but faced technical limitations. VENTIJET is a novel nozzle-based CFV ventilator designed for safe use in critically ill adults. This pilot study evaluated its feasibility and safety in patients with moderate ARDS.

**Methods:**

We conducted a prospective, single-center pilot safety and feasibility study in intubated adults with moderate ARDS and stable oxygenation. After a 1-h baseline period with volume-controlled ventilation (Puritan Bennett™ 840), patients received up to 24 h of VENTIJET ventilation, then returned to conventional ventilation. Primary endpoints were feasibility and safety; secondary exploratory endpoints included gas exchange and respiratory mechanics.

**Results:**

Fourteen patients were enrolled. VENTIJET ventilation was successfully delivered in all participants without protocol interruptions or device-related serious adverse events. After 1 h, PaO₂/FiO₂ was 200 mm Hg compared to 192 mm Hg at baseline (p = 0.090). Over 24 h, oxygenation and respiratory system compliance showed modest improvement, with stable carbon dioxide (CO₂) elimination, transpulmonary pressures, and hemodynamics.

**Conclusions:**

In patients with moderate ARDS, VENTIJET CFV was feasible and safe, maintaining stable gas exchange with modest improvements in respiratory mechanics. These findings support further evaluation in larger, controlled trials, including the full spectrum of ARDS.

*Trial registration* ClinicalTrials.gov Identifier NCT07121257.

**Supplementary Information:**

The online version contains supplementary material available at 10.1186/s40635-026-00876-7.

## Introduction

Acute hypoxemic respiratory failure is a frequent indication of invasive mechanical ventilation in critically ill patients, with acute respiratory distress syndrome (ARDS) representing one of its most severe forms, associated with substantial morbidity and mortality despite advances in supportive care [1–3]. The COVID-19 pandemic further underscored the burden of ARDS, exposing global limitations in ventilator availability and the need for new approaches to support gas exchange while minimizing ventilator-induced lung injury (VILI) [4–6].

Conventional mechanical ventilation alternates inspiratory and expiratory phases through cyclic pressure or volume control. Although effective, these intermittent flow patterns can promote repetitive alveolar collapse and overdistension in heterogeneous lungs, contributing to VILI [7–9]. Continuous-flow ventilation (CFV) approaches represent an alternative paradigm, maintaining a constant gas stream throughout the respiratory cycle. By stabilizing airway pressures and promoting homogeneous lung inflation, CFV may enhance alveolar recruitment, improve oxygenation, and reduce dynamic strain [10–12]. However, earlier CFV systems were limited by high gas consumption, inadequate flow regulation, poor monitoring, and concerns about asynchrony and hyperinflation, preventing widespread clinical adoption [13–15].

Flow-controlled ventilation (FCV) is a distinct ventilatory strategy in which both inspiration and expiration are actively controlled by the ventilator using constant flow profiles in both directions. In FCV, expiration is actively regulated rather than passive, resulting in a near-linear decrease in airway pressure from peak inspiratory pressure to positive end-expiratory pressure (PEEP). In contrast, conventional volume- or pressure-controlled ventilation relies on passive expiration driven by the elastic recoil of the respiratory system. CFV, as implemented in VENTIJET, differs mechanistically in that a constant inlet gas stream is maintained throughout the respiratory cycle, while expiration is modulated through a nozzle-based expiratory flow-braking mechanism rather than through active bidirectional flow control.

Evidence supporting cFCV has progressively expanded from preclinical models to selected human populations. In porcine studies, individualized FCV improved oxygenation while requiring lower minute ventilation compared with pressure-controlled ventilation [16], an ex vivo lung perfusion model demonstrated better oxygenation and a smaller decrease in lung compliance compared with volume-controlled ventilation [11]. In obese patients with healthy lungs, a randomized crossover trial reported that FCV reduced the loss of end-expiratory lung volume and mean lung volume compared with volume-controlled ventilation, consistent with reduced expiratory derecruitment under controlled expiration [12]. In thoracic surgery with one-lung ventilation, a randomized controlled trial found higher PaO₂/FiO₂ under FCV than under pressure-controlled ventilation [17].

However, recent physiological data in ARDS suggest a more complex and potentially safety-relevant response. In a randomized crossover study in moderate-to-severe ARDS, compliance-guided FCV achieved stable gas exchange but showed limited recruitment and a trend toward overdistension—particularly in non-dependent lung regions—leading to early study termination due to safety concerns [18]. Together, these findings indicate that the physiological effects of FCV may vary substantially across phenotypes and settings, and that ARDS-specific evaluation is essential [18].

VENTIJET is a newly developed CFV device specifically designed to address the technical and monitoring constraints that limited the clinical adoption of earlier continuous-flow ventilation systems, including high gas consumption, insufficient monitoring capability, and safety concerns. Building upon the original conceptual work and prototype by Dr. Lucas Picazo [19], VENTIJET allows continuous extratracheal jet ventilation with real-time pressure monitoring, enabling precise adjustment of flow and airway pressures through a compact and mechanically simple design. The system generates an expiratory flow brake via nozzle-driven gas acceleration, aiming to sustain end-expiratory pressure and alveolar recruitment without high plateau pressures. Preclinical evaluations conducted in experimental porcine models (details provided in the Supplemental Material) demonstrated stable gas exchange and respiratory mechanics without evidence of barotrauma or hemodynamic compromise. These findings supported the initiation of a first-in-human clinical investigation to assess the safety and feasibility of CFV using VENTIJET in patients with moderate ARDS.

This pilot, single-center feasibility and safety study aimed to evaluate the tolerability, technical performance, and short-term physiological effects of VENTIJET ventilation in patients with moderate ARDS, establishing the foundation for subsequent randomized controlled studies.

## Methods

### Study design

This was a prospective, single-center pilot safety and feasibility study conducted in the Intensive Care Unit of Hospital del Mar (Barcelona, Spain) between June 2021 and December 2022. The aim of the study was to evaluate the feasibility, safety, and physiological performance of the VENTIJET ventilator, a novel continuous-flow ventilation system specifically developed for clinical use in critically ill patients with acute respiratory distress syndrome (ARDS).

The primary objective was to assess whether the VENTIJET device could be safely implemented and maintained under stable intensive care conditions. Feasibility was defined as the ability to successfully initiate and sustain continuous-flow ventilation for the planned study duration without protocol interruption, while safety was defined as the absence of serious device-related adverse events, hemodynamic instability, or respiratory deterioration requiring discontinuation of the intervention. Secondary objectives included the characterization of gas exchange and respiratory mechanics during VENTIJET ventilation, the evaluation of the technical performance of the system in a clinical setting, and the assessment of patient tolerance and operator usability. These physiological and operational measures were collected to provide preliminary insight into the ventilatory behavior of the device and to inform the design of future controlled studies.

### Ethical and regulatory oversight

The study was approved by the Spanish Agency of Medicines and Medical Devices (AEMPS) and by the institutional ethics committee of Hospital del Mar (reference code 2020/9238), in accordance with Good Clinical Practice and the Declaration of Helsinki. The trial was registered retrospectively on ClinicalTrials.gov (NCT07121257) to ensure transparency in reporting.

VENTIJET Healthcare supplied the ventilator prototype and disposable materials but did not participate in the design of the study, data collection, statistical analysis, or interpretation of the results. Independent external monitoring was performed by the Clinical Research Support Unit (SEIC) of Biocruces Bizkaia to ensure compliance with regulatory and ethical standards.

### Study participants

Adult patients (≥ 18 years) admitted to the Intensive Care Unit (ICU) of Hospital del Mar for acute hypoxemic respiratory failure who met the criteria for moderate ARDS according to the Berlin definition[1] and who did not require prone positioning according to current guidelines[20] (PaO₂/FiO₂ 150–200 mm Hg with PEEP ≥ 5 cm H₂O) were screened for inclusion. Eligible patients were required to have been under invasive mechanical ventilation for at least 48 h with stable oxygenation, defined as less than 10% variation in the PaO₂/FiO₂ ratio and no significant changes in ventilatory settings.

At the time of inclusion, patients still required fully controlled mechanical ventilation due to impaired respiratory system mechanics. Transition to assisted ventilation was considered inappropriate at this stage, as strict control of tidal volume and airway pressures remained necessary. This selection strategy aimed to ensure physiological stability and reduce confounding influences of acute deterioration or evolving lung injury, as well as to avoid including patients in a phase of marked clinical improvement that could act as a confounder for the safety outcomes.

Patients were excluded if they were in the weaning phase of mechanical ventilation, defined by the use of spontaneous breathing modes or ventilatory requirements of FiO₂ ≤ 0.4 or PEEP ≤ 5 cm H₂O. Patients with known obstructive lung disease, including asthma or chronic obstructive pulmonary disease (COPD), were excluded; COPD was defined as previously documented diagnosis supported by pulmonary function testing according to standard diagnostic criteria. Additional exclusion criteria included pregnancy, pneumothorax, bronchopleural fistula, the need for extracorporeal membrane oxygenation or extracorporeal CO₂ removal, and hemodynamic instability requiring high-dose vasoactive support, defined as a norepinephrine infusion exceeding 0.5 µg/kg/min at the time of screening. Patients with a Richmond Agitation–Sedation Scale score greater than –5 or with poor patient–ventilator synchrony despite optimal sedation were also excluded to ensure consistent and controlled ventilation conditions.

Written informed consent was obtained from the patient’s legal representative before enrolment. All participants continued to receive standard-of-care management for ARDS according to institutional protocols, including protective tidal volumes, individualized PEEP titration, and active humidification throughout the study period.

### Device description

VENTIJET is a novel continuous-flow ventilator specifically designed to deliver a constant gas stream throughout the entire respiratory cycle. The system operates by accelerating gas through a calibrated nozzle housed within an expiratory-controlled adapter that functions as a flow-dependent expiratory brake.

**Fig. 1:**
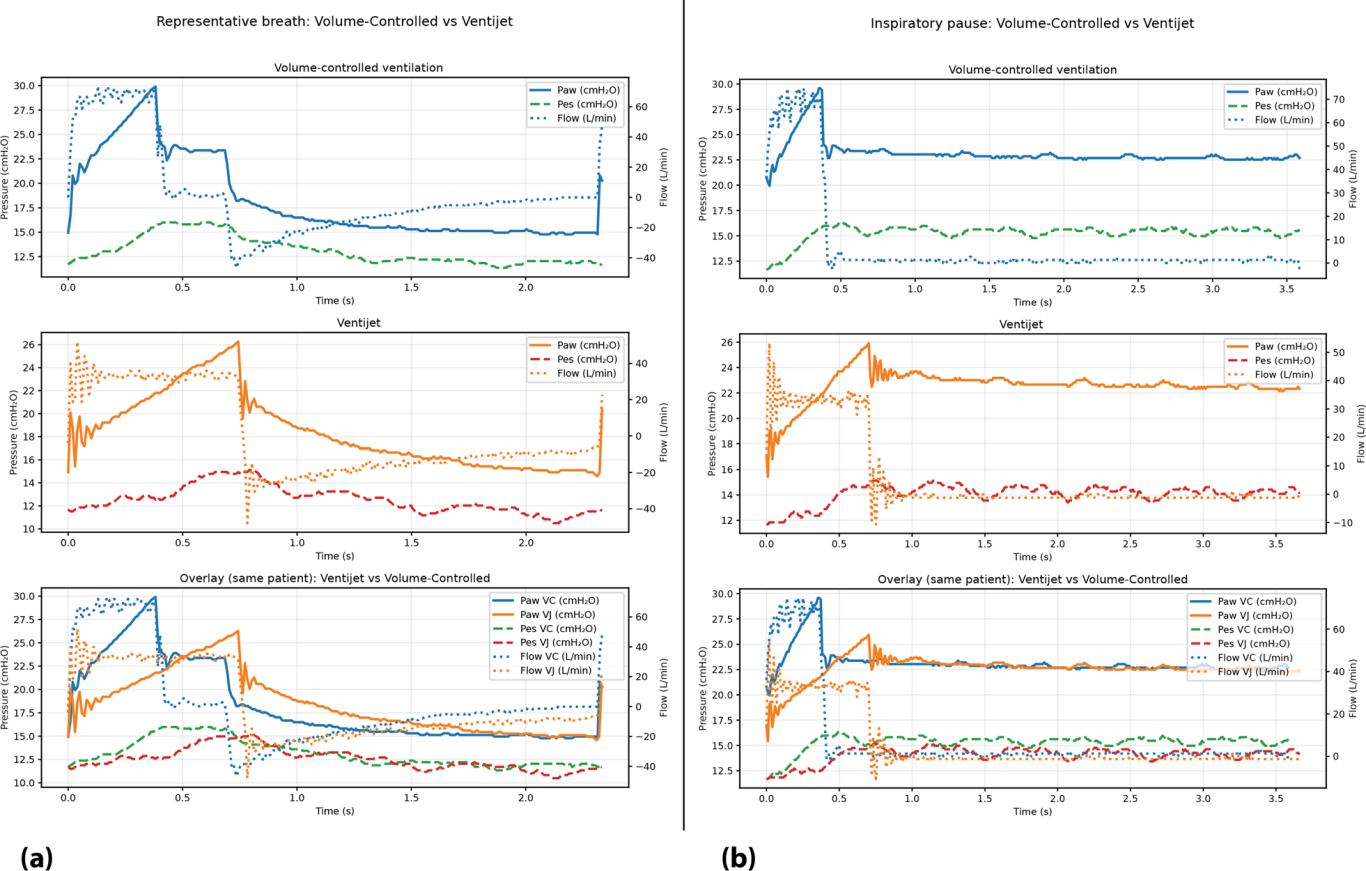
Representative breaths recorded in the same patient during volume-controlled ventilation with the Puritan Bennett™ 840 ventilator (PB840) and continuous-flow ventilation with VENTIJET. **a** Ventilation during regular cycling; **b** measurements obtained during an end-inspiratory hold. Time-aligned airway pressure (Paw), esophageal pressure (Pes), and flow signals are displayed. Volume-controlled ventilation is characterized by a square inspiratory flow pattern with passive expiration, whereas VENTIJET exhibits a continuous inspiratory flow with expiratory flow braking and smoother airway pressure transitions across the respiratory cycle. Signals were recorded at the connection to the endotracheal tube and are shown as raw acquired data

The device allows real-time adjustment of inlet gas flow, inspired oxygen fraction (FiO₂), respiratory rate, and inspiratory time. The transition from inspiration to expiration is time-based, according to the preset inspiratory time and respiratory rate. Pressure and flow are continuously monitored through integrated sensors, and safety features include automatic electronic pressure limitation, disconnection and overpressure alarms, and a mechanical fail–safe valve incorporated into the nozzle system that opens under excessive pressure (> 60 cm H₂O) in case the automatic electronic control system fails. Representative airway pressure and flow-time waveforms illustrating the characteristic continuous inspiratory flow and expiratory flow braking of VENTIJET, compared with volume-controlled ventilation, are shown in Fig. 1.

The conceptual foundation of VENTIJET originates from the early experimental work of Dr. Lucas Picazo, whose doctoral thesis first described the principles of continuous-flow extratracheal jet ventilation principles later adapted in the current VENTIJET system [19].

**Fig. 2:**
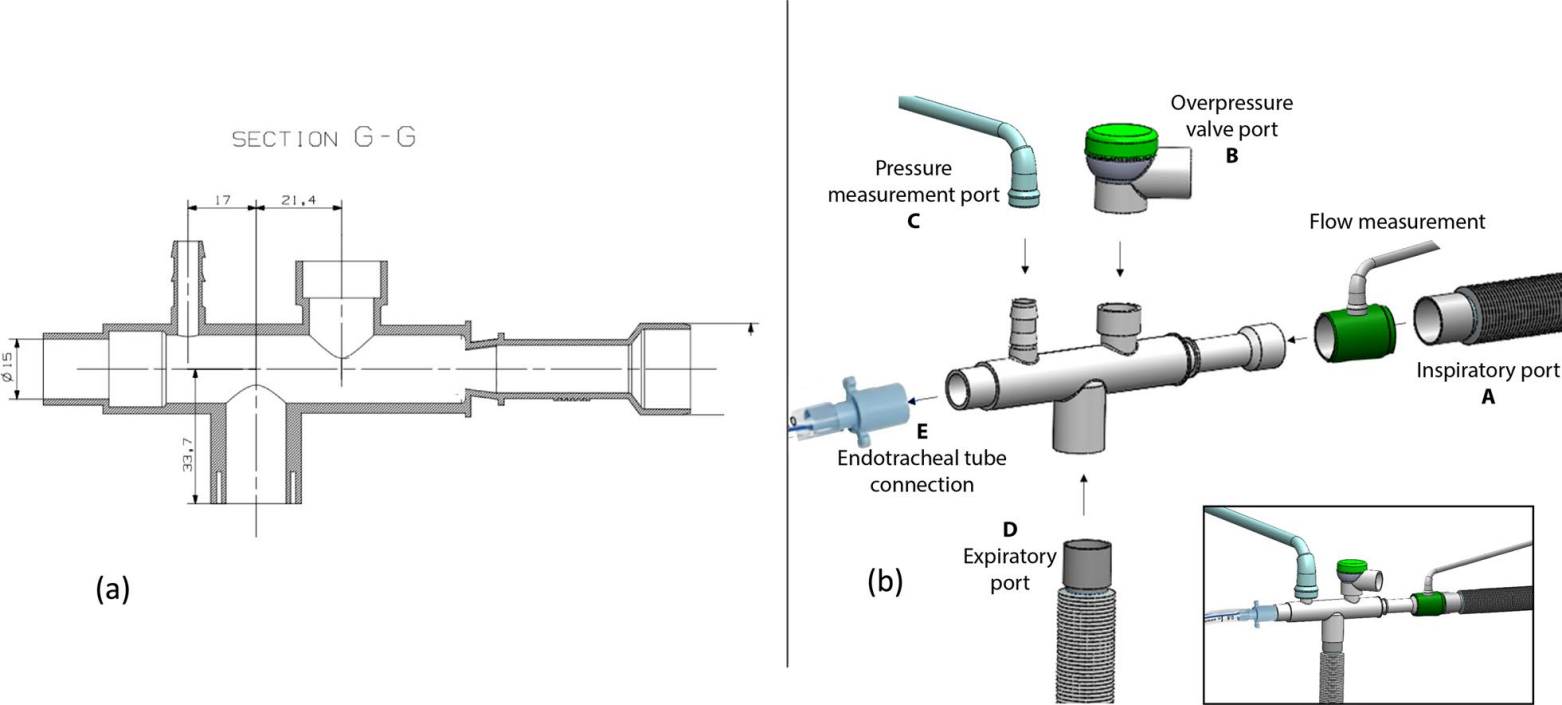
**a** Technical cross-sectional view of the VENTIJET nozzle, illustrating its internal geometry and gas flow pathway. **b** Schematic representation of the ECAF (expiratory-controlled adapter for flow), highlighting its functional ports: A—inspiratory port, B—overpressure valve port, C—pressure measurement port, D—expiratory port, E—endotracheal tube connection. The ECAF houses the VENTIJET nozzle and serves as the interface between the control unit and the patient

**Fig. 3:**
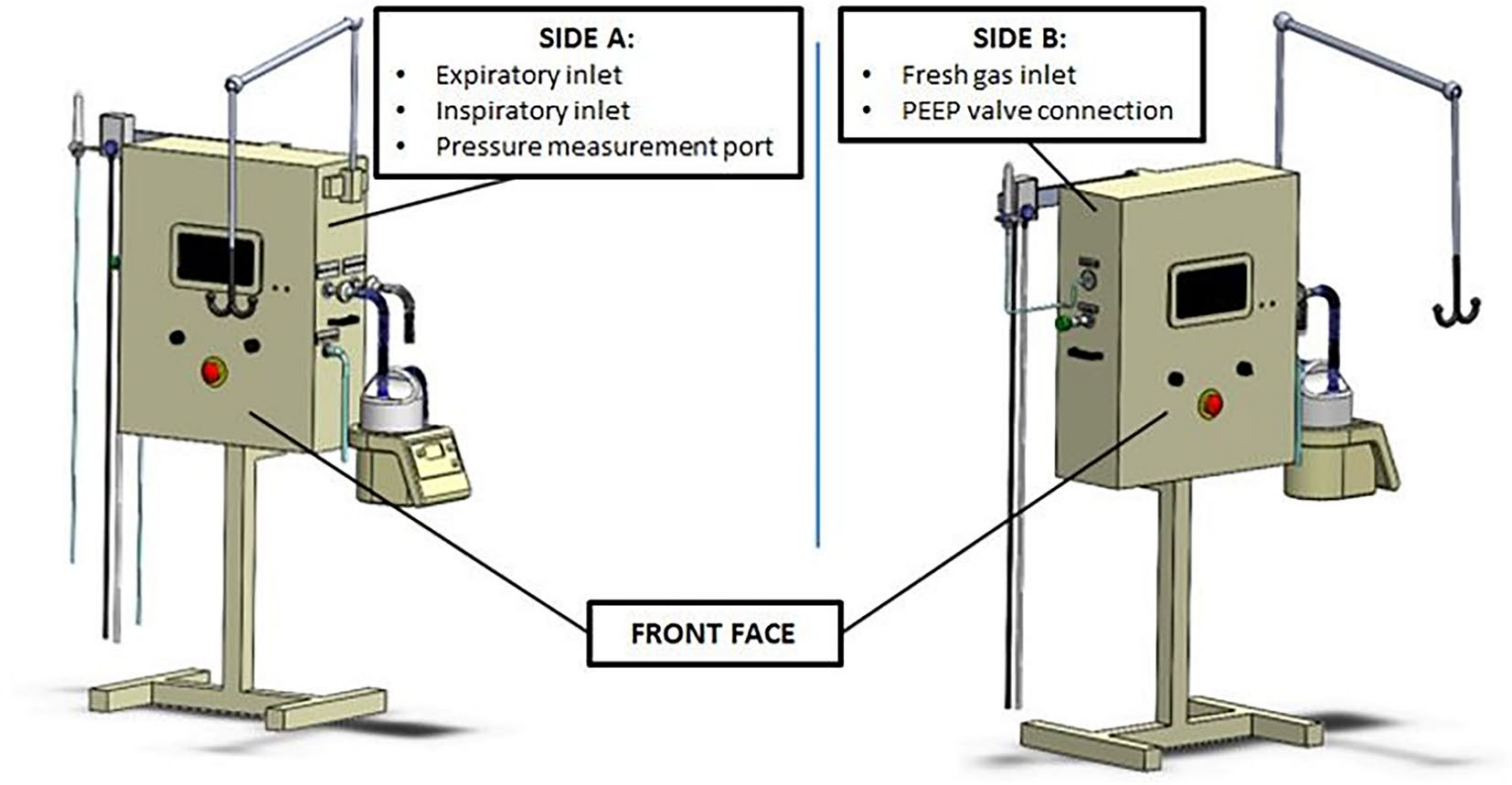
Schematic representation of external view of the VENTIJET ventilator

Schematic representations of the device and nozzle are provided in Figs. 2 and 3, with additional technical details available in the Supplementary Appendix.

### Study procedures

Before the start of each intervention, both the VENTIJET device and the external monitoring system (CARESCAPE B650, GE Healthcare, Chicago, IL, USA) were calibrated to ensure synchronized data acquisition and accurate measurement of airway pressure and flow. All patients were sedated and paralyzed according to standard intensive care protocols to maintain controlled ventilation and ensure the absence of spontaneous respiratory effort. Active humidification was provided throughout the study using a heated humidifier.

At baseline, patients were ventilated with a conventional volume-controlled mode using a Puritan Bennett 840 ventilator (Medtronic, Minneapolis, MN, USA). Ventilatory parameters were optimized following a lung-protective strategy, including a tidal volume ≤ 6 mL/kg predicted body weight, PEEP titrated to achieve an end-expiratory transpulmonary pressure between 0 and + 2 cm H₂O, respiratory rate adjusted to maintain pH > 7.30, and FiO₂ tailored to keep peripheral oxygen saturation ≥ 92%.

Once physiological stability was confirmed, patients were transitioned to VENTIJET ventilation using an end-expiratory clamping technique to prevent alveolar derecruitment. VENTIJET inlet flow was adjusted to reproduce the same tidal volume as during the preceding volume-controlled ventilation, while inspiratory time was set according to the inspiratory time prescribed during volume-controlled ventilation and rounded to the nearest tenth, as permitted by the device, and verified by external monitoring. An arterial blood gas sample and a full set of ventilatory and hemodynamic variables were collected after 1 h of VENTIJET ventilation. If no adverse events occurred, ventilation with VENTIJET continued for up to 24 h, with predefined evaluations at 6, 12, and 24 h.

**Fig. 4:**
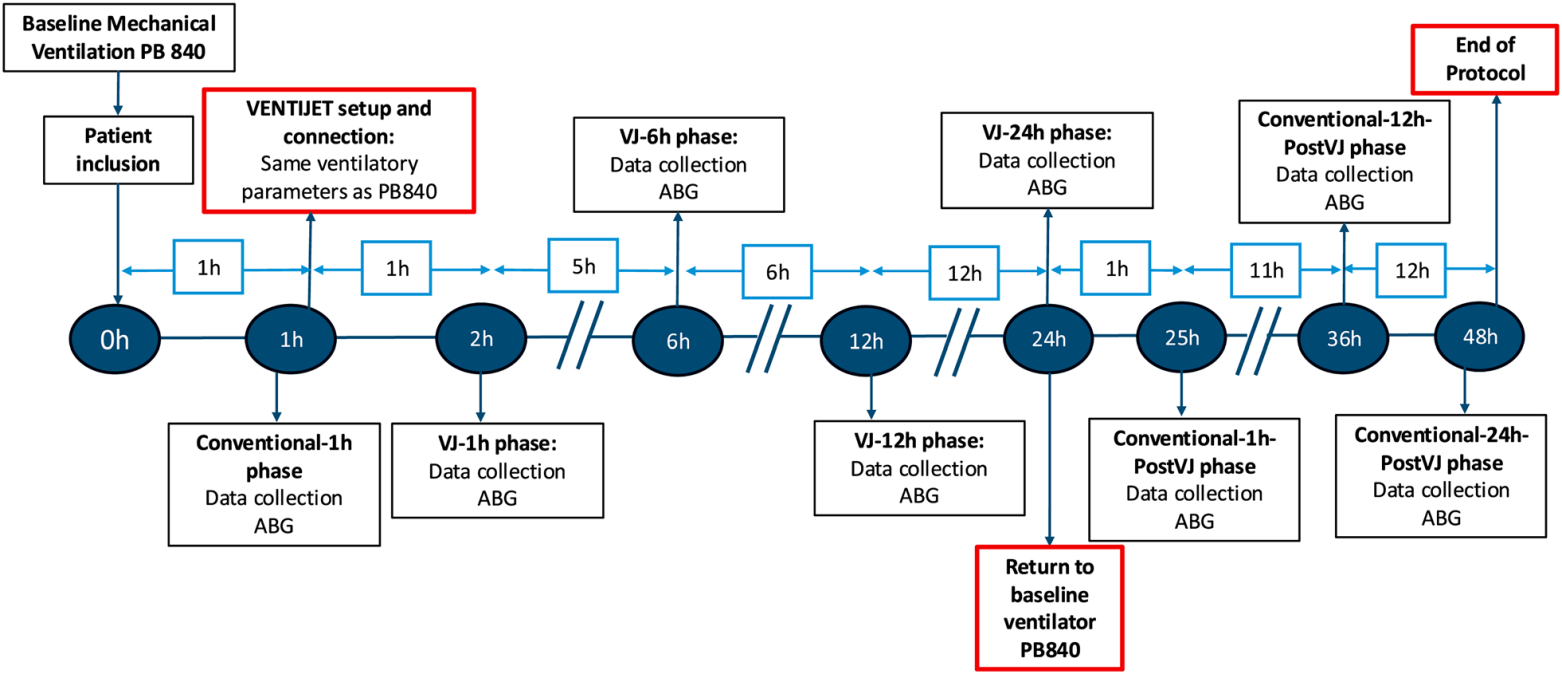
Study protocol timeline. After confirming eligibility, patients remained on volume-controlled ventilation with the Puritan Bennett™ 840 (PB840) for a 1-h PB840 phase. They were then transitioned to the VENTIJET ventilator and maintained on it for up to 24 h in the absence of adverse events. Arterial blood gas (ABG) samples and respiratory and hemodynamic variables were collected at predefined intervals during VENTIJET use (1 h, 6 h, 12 h, and 24 h). After this period, patients were reconnected to the PB840, and further data were collected at 1 h, 12 h, and 24 h after reconnection (post-VJ phase). *ABG* arterial blood gas, *PB840* Puritan Bennett™ 840 ventilator, *VJ* VENTIJET, *Post-VJ* post-VENTIJET reconnection.

At the end of the intervention period, patients were reconnected to the conventional ventilator for ongoing standard care. Additional arterial blood gases and physiological measurements were collected 1, 12, and 24 h after returning to conventional ventilation. The study flow and timing of assessments are summarized in Fig. 4.

### Measurements and outcomes

The primary endpoints of the study were feasibility and safety. Feasibility was defined as the successful initiation and maintenance of continuous-flow ventilation using the VENTIJET device for the planned study duration under stable clinical conditions, without unplanned protocol interruption. Safety was defined as the absence of serious device-related adverse events, including barotrauma, hemodynamic instability, or sustained desaturation requiring discontinuation of the intervention.

Secondary outcomes focused on physiological performance and included variables related to gas exchange and respiratory mechanics. Arterial blood gases were obtained at each predefined timepoint to assess oxygenation (PaO₂, PaO₂/FiO₂ ratio) and carbon dioxide elimination (PaCO₂, pH, HCO₃⁻). Ventilatory variables (such as tidal volume, respiratory rate, minute ventilation, inspiratory time, PEEP, and FiO₂) were continuously monitored and recorded. Respiratory system mechanics were evaluated using airway and transpulmonary pressures obtained with a nasogastric balloon catheter, following standard validation and occlusion techniques as previously described [21]. Compliance of the respiratory system, chest wall, and lung were calculated using these measurements [22].

Hemodynamic parameters, including mean arterial pressure, heart rate, and vasoactive drug dose, were monitored continuously. Lactate concentration and peripheral oxygen saturation were also recorded as complementary indicators of tissue oxygenation and metabolic response.

All data were collected electronically and verified by the external monitoring system (CARESCAPE B650, GE Healthcare, Chicago, IL, USA) to ensure consistency and accuracy. The timing of measurements was identical for all participants and adhered to the predefined protocol schedule.

### Safety monitoring

An investigator remained at the bedside throughout each VENTIJET intervention to ensure continuous observation and the ability to respond immediately to any clinical change. Standard intensive care monitoring was maintained at all times, including continuous electrocardiography, invasive arterial pressure, pulse oximetry, and capnography. All adverse events were prospectively defined and classified according to severity and relatedness to the study device or procedure.

A protocol-defined list of safety criteria mandated immediate discontinuation of VENTIJET ventilation and reconnection to the conventional ventilator. These criteria included hemodynamic instability, defined as mean arterial pressure < 65 mm Hg despite vasoactive support; oxygen desaturation < 88% sustained for more than 2 minutes; unexpected hypercapnia with pH < 7.20; and any suspicion of barotrauma pneumothorax or subcutaneous emphysema.

All adverse and serious adverse events were reported within 24 h to the independent Clinical Research Support Unit of Biocruces Bizkaia, which oversaw safety adjudication and regulatory compliance. Device malfunctions, user errors, and interruptions related to technical performance were documented separately.

No changes in sedation strategy, ventilatory targets, or supportive therapies were introduced by the protocol. The treating medical team maintained full authority over all clinical decisions, ensuring that participation in the study did not interfere with standard patient management.

### Statistical analysis

Given the exploratory, pilot nature of the study, all analyses were descriptive and focused on feasibility, safety, and physiological trends over time. Continuous variables are reported as medians with interquartile ranges, and categorical variables as counts and percentages. Changes across study timepoints were examined using paired non-parametric tests for repeated measures (Friedmann or Wilcoxon tests as appropriate).

Physiological data were summarized graphically to illustrate temporal evolution of key parameters, including PaO₂/FiO₂ ratio, respiratory system compliance, and driving pressures. Missing data were not imputed, as measurements followed a fixed schedule and each participant served as their own control under standardized ventilatory conditions.

The sample size of 14 patients was determined pragmatically to allow initial evaluation of device feasibility and to identify potential safety concerns in a clinical environment. Statistical analyses were performed using Python 3.11 (Python Software Foundation, Wilmington, DE) with the SciPy package, and SPSS version 18 (IBM Corp., Armonk, NY, USA).

## Results

### Study population

A total of 14 patients were enrolled between June 2021 and December 2022. All participants met criteria for moderate ARDS according to the Berlin definition and completed the planned intervention without protocol deviations. No patient was excluded after enrolment, and all were included in the final analysis.

The median age of participants was 64 years (57–71), and nine patients (64%) were male. The predominant cause of respiratory failure was viral pneumonia (64%), most commonly related to SARS-CoV-2 infection. Most patients had at least one comorbidity, including arterial hypertension (64%), immunosuppression (50%), and chronic kidney disease (36%). The median PaO₂/FiO₂ ratio at inclusion was 191 mm Hg (174–212), consistent with moderate ARDS.

At baseline, patients had been under invasive mechanical ventilation for a median of 2 days (2–9) with lung-protective settings, including a median tidal volume of 5.9 mL/kg predicted body weight (5.6–6.4) and PEEP of 14 cm H₂O (13–15). Sedation and neuromuscular blockade were used in accordance with local practice, with 11 patients (79%) receiving continuous neuromuscular blockade at the time of inclusion.

All patients successfully transitioned to VENTIJET ventilation and completed the 24-h protocol phase. Baseline characteristics of the study population are detailed in Table 1.

**Table 1:** Baseline characteristics of patients at inclusion

Category	Variable	*p *value
Demographics	Age (years)	64 (57–71)
	Gender (male)	9 (64.3)
	Actual body weight (kg)	83.0 (60.8–90.0)
	Ideal body weight (kg)	59.6 (57.4–68.2)
	Height (cm)	164 (160–170)
	BMI (kg/m^2^)	28.8 (25.3–34.3)
Clinical history	Diagnosis (n, %)	
	Bacterial pneumonia Streptococcus pneumoniae Legionella pneumophila Escherichia coli Bordetella bronchiseptica	5 (35.7) 2 (14.3) 1 (7.1) 1 (7.1) 1 (7.1)
	Viral pneumonia SARS-CoV-2 Influenzae A	9 (64.3) 7 (50.0) 2 (14.3)
	Comorbidities (n, %):	
	Smoking	8 (57.1)
	Arterial hypertension	9 (64.3)
	Diabetes mellitus	4 (28.6)
	Chronic kidney disease	5 (35.7)
	Hemodialysis	1 (7.1)
	Active neoplastic disease	2 (14.3)
	Liver cirrhosis	1 (7.1)
	Transplantation	4 (28.6)
	Immunosuppression	7 (50.0)
Ventilatory variables	Best PaO_2_/FiO_2_ ratio pre-inclusion (mm Hg)	191 (174–212)
	Tidal volume (V_T_, ml)	365 (330–380)
	V_T_/predicted body weight (ml/kg)	5.96 (5.61–6.45)
	Respiratory rate (rpm)	26 (24–28)
	Minute volume (MV) (l/min)	8.80 (8.35–9.89)
	Inspiratory time (sec)	0.72 (0.66–0.83)
	Inspiratory flow (l/min)	60 (60–60)
	PEEP (cmH2O)	14 (13–15)
	FiO_2_ (%)	0.48 (0.40–0.50)
ABG (arterial blood gas) at inclusion	pH	7.41 (7.36–7.43)
	PaCO_2_ (mm Hg)	41.6 (36.2–49.8)
	PaO_2_ (mm Hg)	81.2 (72.4–92.4)
	PaO_2_/FiO_2_ (mm Hg)	181 (172–193)
	Bicarbonate (mmol/l)	25.3 (22.4–29.3)
	SaO2 (%)	96.2 (94.3–97.2)
	CaO2 (ml/dl)	12.2 (11.0–13.1)
Clinical situation at inclusion	MAP (mm Hg)	73.0 (68.5–79.2)
	HR (bpm)	76 (68–96)
	SpO2 (%)	95 (94–96)
	Noradrenaline (ug/kg/min)	0.07 (0.00–0.25)
	APACHE-II (score)	17 (13–23)
	Previous prone position (n, %)	6 (43)
	Days of invasive mechanical ventilation before Ventijet	2 (2–9)
	Corticosteroids (n, %)	7 (50)
	Neuromuscular blockade (NMB) (n, %)	11 (80)

Data are expressed as median (IQR) or n(%). *ABG* arterial blood gas, *APACHE-II* Acute Physiology and Chronic Health Evaluation II score, *BMI* body mass index, *CaO₂* arterial oxygen content, *FiO₂* fraction of inspired oxygen, *HR* heart rate, *MAP* mean arterial pressure, *MV* minute volume, *NMB* neuromuscular blockade, *PaCO₂* partial pressure of carbon dioxide, *PaO₂* partial pressure of oxygen, *PEEP* positive end-expiratory pressure; rpm: respiratory rate per minute, *SaO₂* arterial oxygen saturation, *SpO₂* peripheral oxygen saturation, *Vt* tidal volume

### Feasibility

VENTIJET ventilation was successfully initiated and maintained in all 14 participants (100%), fulfilling the predefined feasibility criteria. The transition from conventional ventilation to VENTIJET was achieved without hemodynamic instability or oxygen desaturation in any case.

Continuous-flow ventilation was sustained for the full 24-h intervention period in all cases. No unplanned protocol interruptions occurred, and all sessions were completed as scheduled. Device performance remained stable throughout the study, with no episodes of unexpected shutdown, flow irregularity, or pressure instability. No recalibration or manual over-ride was required after initial setup.

Bedside staff reported no technical difficulties related to connection, monitoring synchronization, or alarm management. The device consistently delivered a steady gas flow without fluctuations in airway pressure, and all monitored parameters remained within expected physiological ranges. Overall, these findings demonstrate that VENTIJET ventilation was technically feasible and operationally stable under intensive care conditions.

### Safety

No serious adverse events related to the VENTIJET device or its operation occurred during the study. All patients completed the planned 24-h intervention period without meeting any protocol-defined criteria for discontinuation. Continuous bedside observation was maintained throughout each session, and no episodes of barotrauma, pneumothorax, or hemodynamic collapse were observed.

Three mild adverse events were reported. One patient experienced transient desaturation following endotracheal suctioning, which resolved immediately without ventilatory adjustments. Another—who had undergone two prone-positioning sessions prior to study enrolment and had stabilized in the supine position for 2 days—developed recurrent hypoxemia during the study period, requiring a new prone-positioning session with subsequent improvement in oxygenation. No further prone maneuvers were required after the study. A third patient experienced brief derecruitment following disconnection for secretion clearance, which was promptly reversed by transiently increasing PEEP and FiO₂. None of these events required interruption of the VENTIJET session or were considered device-related. Details about the adverse events are provided in the supplemental material.

No patient required escalation of respiratory support, or rescue therapies, and no unexpected alterations in heart rate, arterial pressure, or vasoactive drug requirements occurred during or after the intervention. The independent monitoring committee reviewed all events and confirmed that no serious or unexpected device-related adverse events were identified.

### Physiological observations

VENTIJET ventilation was associated with stable gas exchange and respiratory mechanics throughout the study period. After 1 h of continuous-flow ventilation, the median PaO₂/FiO₂ ratio was 200 mm Hg (177–224), closely matching the values observed during conventional ventilation 1 h prior to VENTIJET initiation (192 mm Hg [163–215]; p = 0.090). Carbon dioxide elimination remained within physiological limits, with a median PaCO₂ of 42 mm Hg (39–53) and a pH of 7.40 (7.32–7.40).

During the 24-h intervention phase, both oxygenation and respiratory system compliance demonstrated gradual improvement. The highest PaO₂/FiO₂ ratio observed during VENTIJET ventilation reached 231 mm Hg (206–258), compared with 209 mm Hg (184–221) in the preceding conventional phase, p = 0.009. Similarly, the maximum median respiratory system compliance increased from 29 mL/cm H₂O (23–51) during the pre-VENTIJET phase to 34 mL/cm H₂O (25–43) during VENTIJET ventilation (p = 0.014). This improvement was paralleled by an increase in lung compliance from 40 mL/cm H₂O (33–59) during the preceding conventional ventilation phase to 51 mL/cm H₂O (42–79) during VENTIJET use (p = 0.002). These effects were maintained after returning to conventional ventilation.

Inspiratory and expiratory transpulmonary pressures remained within safe physiological ranges. Median inspiratory transpulmonary pressure was 7.4 cm H₂O (4.1–9.0), and expiratory transpulmonary pressure was 0.5 cm H₂O (–2.3–2.6). Lactate concentration decreased modestly during VENTIJET ventilation [from 1.76 mmol/L (1.19–2.20) at baseline to 1.36 mmol/L (1.04–1.74)].

Hemodynamic parameters (heart rate, mean arterial pressure, and vasoactive drug requirements) remained unchanged. Minute ventilation, tidal volume, and driving pressure also remained stable across the intervention period.

**Fig. 5:**
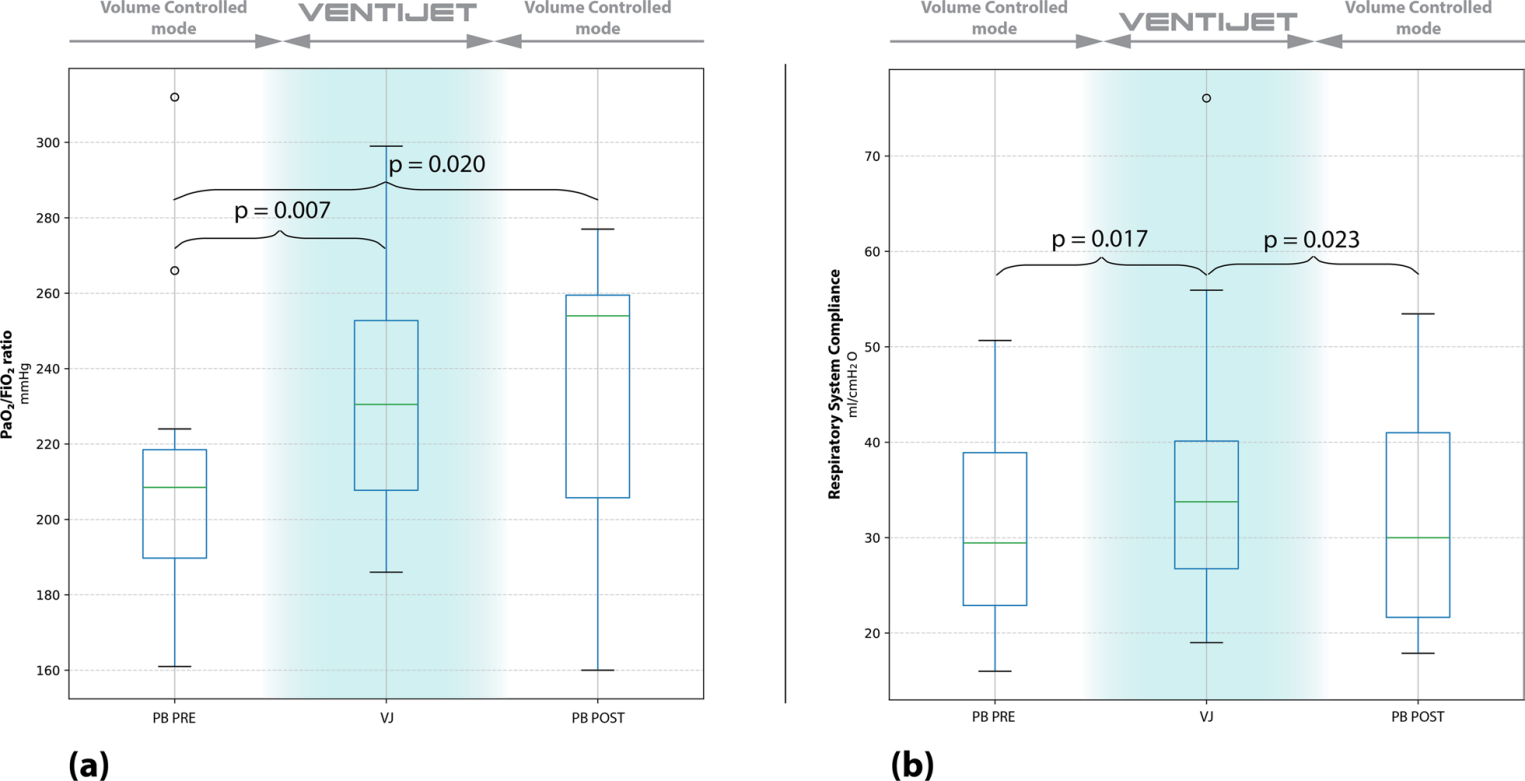
Maximum PaO₂/FiO₂ ratio and respiratory system compliance (Crs) across study phases.** a** Boxplots showing the highest PaO₂/FiO₂ ratio recorded within each 24-h period: the 24 h prior to VENTIJET ventilation (PB PRE), the 24-h VENTIJET phase (VJ), and the 24 h following VENTIJET discontinuation (PB POST). **b** Boxplots displaying the maximum Crs values observed within the same time intervals. Statistical comparisons reflect paired differences between phases. VENTIJET refers to the continuous-flow ventilation period using the VENTIJET device

Data comparing conventional ventilation with the first hour of VENTIJET ventilation are summarized in Table 2. The temporal evolution of oxygenation and respiratory system compliance across the pre-VENTIJET, during VENTIJET and post-VENTIJET phases is illustrated in Fig. 5.

**Table 2:** Comparison of ventilatory, pulmonary mechanics, and clinical parameters after 1 h of ventilation with PB840 and VENTIJET with same tidal volume

Category	Variable	PB 840 1 h	Ventijet 1 h	*p* value
Ventilatory Variables	V_T_ (ml)	305 (290–333)	305 (290–340)	0.317
	V_T_/PBW (ml/kg)	5.14 (4.74–5.48)	5.14 (4.74–5.48)	0.655
	RR (rpm)	26 (24–29)	26 (24–29)	1.000
	MV (l/min)	8.24 (7.31–8.52)	8.28 (7.31–8.69)	0.317
	Inspiratory time (sec)	0.73 (0.66–0.81)	0.75 (0.70–0.83)	0.010
	Inspiratory flow (l/min)	60 (60–60)	28 (24–30)	0.001
	PEEP (cmH_2_O)	15.0 (13.8–16.3)	14.5 (13.8–16.3)	0.180
	FiO_2_ (%)	0.45 (0.38–0.48)	0.45 (0.38–0.48)	0.317
Pulmonary mechanics	Insp_aw_ pressure (cm H_2_O)	25.4 (23.4–29.2)	25.0 (23.3–27.0)	0.198
	Esp_aw_ pressure (cm H_2_O)	15.1 (13.1–16.6)	14.8 (13.3–16.1)	0.061
	Insp_es_ pressure (cm H_2_O)	17.4 (16.0–20.1)	18.2 (16.6–21.1)	0.084
	Esp_es_ pressure (cm H_2_O)	14.1 (12.5–15.8)	14.8 (12.8–15.7)	0.084
	Insp_PL_ pressure (cm H_2_O)	8.4 (5.7–11.4)	7.4 (4.1–9.0)	0.005
	Esp_PL_ pressure (cm H_2_O)	1.3 (-0.6–2.8)	0.5 (-2.3–2.6)	0.016
	DP_aw_ (cm H_2_O)	11.5 (8.3–13.3)	10.6 (8.6–13-1)	0.331
	DP_es_ (cm H_2_O)	3.8 (2.6–4.4)	3.3 (2.9–4.4)	0.675
	DP_L_ (cm H_2_O)	7.9 (5.3–10.3)	7.2 (5.2–7.9)	0.084
	C_rs_ (ml/cm H_2_O)	26.9 (22.6–40.6)	30.9 (21.1–39.2)	0.198
	C_s_ (ml/cm H_2_O)	80 (66–114)	89 (69–116)	0.556
	C_L_ (ml/cm H_2_O)	37 (31–59)	46 (38–67)	0.084
Arterial blood gas (ABG) variables	pH	7.39 (7.34–7.41)	7.40 (7.32–7.40)	0.959
	PaCO_2_ (mm Hg)	45.3 (39.4–51.0)	42.3 (39.2–53.3)	0.470
	PaO_2_ (mm Hg)	78.3 (75.5–97.6)	85.1 (77.1–91.5)	0.109
	PaO_2_/FiO_2_ (mm Hg)	192 (163–215)	200 (177–224)	0.090
	SaO_2_ (%)	95.6 (94.5–96.9)	96.2 (94.9–96.8)	0.173
	CaO_2_ (ml/dl)	12.1 (11.3–13.0)	12.1 (11.1–13.1)	0.158
	Bicarbonate (mmol/l)	24.1 (21.6–28.4)	25.7 (20.6–28.4)	0.615
	Lactate (mmol/L)	1.76 (1.19–2.20)	1.36 (1.04–1.74)	0.008
Clinical parameters	MAP (mmHg)	74 (70–81)	76 (80–72)	0.889
	HR (bpm)	78 (71–96)	76 (72–93)	0.221
	SpO_2_ (%)	95.0 (94.0–97.3)	95.5 (94.0–96.0)	0.914
	etCO_2_ (mm Hg)	34 (29–41)	34 (30–41)	0.396
	GapCO_2_ (mm Hg)	10.9 (6.4–12.8)	9.7 (8.7–12.9)	0.551
	Noradrenaline (ug/kg/min)	0.07 (0.00–0.25)	0.08 (0.00–0.24)	0.285

All pulmonary mechanics were determined from measurements obtained during inspiratory and expiratory hold maneuvers. Values are expressed as median (IQR)

*Vt* tidal volume, *PBW* predicted body weight, *RR* respiratory rate, *MV* minute volume, *PEEP* positive end-expiratory pressure, *FiO₂* fraction of inspired oxygen, *etCO₂* end-tidal carbon dioxide, *GapCO₂* arterial–end-tidal CO₂ gradient, *Inspaw/Espaw* airway pressures, *InspPL/EspPL* transpulmonary pressures, *DPaw, DPes, DPL* driving pressures (airway, esophageal, transpulmonary), *Crs* compliance of the respiratory system, *Cs* chest wall compliance, *CL* lung compliance, *PaCO₂/PaO₂* partial pressures of CO₂/O₂, *SaO₂/SpO₂* arterial/peripheral oxygen saturation, *CaO₂* arterial oxygen content, *MAP* mean arterial pressure, *HR* heart rate

## Discussion

This pilot safety and feasibility study demonstrates that continuous-flow ventilation with the VENTIJET system is both feasible and safe in critically ill patients with moderate ARDS. The intervention was successfully completed in all participants, with no adverse events attributable to the device and no need for protocol interruption. Throughout the 24-h intervention, VENTIJET maintained stable gas exchange and mechanical performance, supporting its short-term tolerability in a controlled ICU setting.

Although VENTIJET shares conceptual similarities with other controlled-flow ventilation strategies, such as flow-controlled ventilation (FCV), these approaches are not interchangeable. Recent physiological studies of FCV in moderate-to-severe ARDS have shown that while controlled expiration may redistribute ventilation toward dependent lung regions, compliance-guided FCV can also increase tidal volumes, transpulmonary pressures, and mean airway pressure, resulting in overdistension of non-dependent regions and raising safety concerns [16, 18]. Taken together, these observations highlight that the physiological impact of expiratory flow modulation in ARDS may depend strongly on the specific mechanical implementation of each strategy, and that findings obtained with FCV should not be directly extrapolated to other approaches. Within this framework, VENTIJET represents an alternative mechanical implementation, relying on a nozzle-based expiratory flow-braking mechanism that sustains end-expiratory pressure without active intratracheal pressure-guided expiration or compliance-guided escalation of driving pressures. These mechanistic differences may partly explain the lower transpulmonary pressures observed with VENTIJET in this study. Consequently, the physiological effects observed here should be interpreted as specific to the VENTIJET system and its implementation, rather than extrapolated to controlled-flow ventilation (CFV) strategies as a whole.

Although the study was not designed or powered to evaluate physiological efficacy, the physiological behavior observed during CFV was broadly consistent with the expected effects of maintaining a constant gas stream throughout the respiratory cycle. This pattern (characterized by a stable mean airway pressure and reduced cyclic variation) may theoretically minimize alveolar collapse and mechanical strain compared with conventional volume- or pressure-controlled ventilation [7, 8, 23, 24]. We acknowledge that, at a macroscopic waveform level, the differences in expiratory airway pressure and flow between volume-controlled ventilation and VENTIJET appear modest in Fig. 1. The hypothesized relevance of CFV may not depend on large changes in peak pressures, but rather on modulation of expiratory flow dynamics, smoothing of pressure transitions, and potential reduction of abrupt alveolar deflation patterns. The modest improvement in oxygenation and respiratory system compliance observed during VENTIJET use, together with preserved carbon dioxide clearance, are compatible with a possible signal of enhanced alveolar recruitment without increasing dynamic hyperinflation or driving pressure. However, given the non-randomized and time-sequential design of the intervention, these findings should be interpreted as exploratory and cannot establish a causal relationship between the ventilatory mode and the observed physiological changes.

Our observations are consistent with prior experimental work describing the potential benefits of flow-controlled or continuous-flow ventilation modes in mitigating VILI. Preclinical studies have shown that steady flow delivery can improve gas distribution and reduce regional overdistension compared with conventional modes [10–12]. Earlier systems, however, faced technical limitations related to high gas consumption and limited monitoring capabilities, which hindered clinical translation [13–15]. VENTIJET was specifically designed to address these constraints by combining precise real-time monitoring, allowing continuous ventilation to be applied safely in adult ICU patients under controlled conditions.

By maintaining constant flow throughout the entire respiratory cycle, CFV may promote a more homogeneous pattern of alveolar inflation [23–26]. The modest improvement in oxygenation and respiratory system compliance observed during VENTIJET use is consistent with this physiological rationale. However, given the pilot, non-randomized, and time-sequential design of the study, these findings should be interpreted cautiously and cannot be taken as evidence of reduced regional overdistension or cyclic strain.

Another relevant observation (although beyond the primary scope of this feasibility and safety study) is that CFV appeared to be well-tolerated from a hemodynamic standpoint. Despite the modest increase in mean airway pressure inherent to this mode, no patient developed hypotension or required escalation of vasoactive support. Within the limits of this pilot design, this suggests the increase in mean airway pressure did not translate into clinically significant rises in intrathoracic pressure or reductions in venous return. The absence of dynamic hyperinflation or gas trapping, as reflected by stable transpulmonary pressures and end-tidal CO₂, further supports the mechanical tolerability of the system in this controlled setting.

These physiological characteristics may hold potential relevance for ARDS management. Low tidal volume ventilation often results in increased atelectasis and cyclic alveolar reopening [23–26]. Continuous-flow, by providing smoother transitions between inspiration and expiration, could theoretically help maintain alveolar recruitment without increasing driving pressure, thereby lowering mechanical energy applied to the lung parenchyma [10–12]. These hypotheses, however, require formal testing in studies specifically designed to assess physiological and clinical efficacy.

Several limitations must be acknowledged. First, the small sample size inherent to a pilot safety and feasibility study restricts generalizability and limits statistical interpretation. Second, patients were included at the milder end of the ARDS spectrum; therefore, these findings cannot be extrapolated to more severe forms of ARDS. Nonetheless, the primary objective of this early phase trial was to evaluate feasibility and safety rather than efficacy, and the study met these aims.

Third, although active humidification was provided throughout all study phases, the efficiency of humidification during CFV was not formally assessed and warrants further investigation. Future studies should also include more comprehensive monitoring of gas consumption, flow dynamics, and potential effects on secretion clearance. While these aspects did not raise safety concerns in this trial, they will be important for understanding the practical integration of CFV into routine ICU practice.

Finally, because this trial was conducted under highly controlled conditions in deeply sedated, fully passive patients, the applicability of these findings to patients with spontaneous respiratory effort remains uncertain.

## Conclusion

In this pilot safety and feasibility study, continuous-flow ventilation with the VENTIJET system was delivered safely and consistently in critically ill patients with moderate ARDS, maintaining stable gas exchange and demonstrating a favorable short-term mechanical profile over 24 h. These findings support further evaluation of this device in larger, controlled clinical studies to assess physiological and clinical outcomes and to better define its potential role within lung-protective ventilation strategies for ARDS.

## Supplementary Information

Additional file 1.

## Data Availability

The data sets generated and analyzed during the current study are not publicly available due to patient confidentiality constraints, but are available from the corresponding author upon reasonable request.
