# Supplementary material for: Continuous-flow ventilation with VENTIJET in moderate ARDS: a pilot safety and feasibility study
Source: Intensive Care Med Exp. 2026 Apr 3;14:42. doi: 10.1186/s40635-026-00876-7 (PMC13046885; doi:10.1186/s40635-026-00876-7)
Supplement: Supplementary file 1 — Additional file 1. [file 40635_2026_876_MOESM1_ESM.docx]

Supplemental Online Material

**Continuous-flow ventilation with VENTIJET in moderate ARDS: a pilot safety and feasibility study**

Lucía Picazo Moreno

Andrea Castellví-Font

Maria Acer Puig

Carles Camañes Mayordomo

Cristina Soriano Rodríguez

Joan Ramon Masclans Enviz

Francisco José Parrilla-Gómez

1. **Historical Rationale and Conceptual Development of VENTIJET**

The development of the VENTIJET system was inspired by the work of Dr. Lucas Picazo, an intensivist who in the early 1990s sought alternative ventilatory strategies for patients with severe respiratory failure. At a time when mechanical ventilation predominantly relied on volume-oriented modes, often associated with high rates of barotrauma and oxygen toxicity, Dr. Picazo proposed an alternative hybrid approach combining continuous extratracheal jet ventilation with pressure-controlled ventilation (15).

Supplementary **Figure 1** shows a schematic from Dr. Picazo’s original doctoral thesis, detailing the conceptual basis and key components of the VC-ET circuit.

Supplementary **Figure 2** displays the first prototype developed in the 1990s, including the manually adjustable flow controls, jet nozzle system, and pressure monitoring devices used in preliminary bench and clinical testing.

This hybrid technique was tested in a cohort of 21 patients with acute respiratory distress syndrome (ARDS), demonstrating improvements in oxygenation and lung aeration without increasing complications. His doctoral thesis, completed in 1997 and partially funded by the Junta de Andalucía (Exp. 166/96), described in detail the physiological rationale and radiological findings supporting the technique (15). The ventilator was patented in Spain under Patent No. P9601844.

The current VENTIJET system builds upon those initial concepts and technical principles. The core idea—delivering continuous high-flow oxygen through a supraglottic catheter to increase alveolar volume while minimizing airway pressure—remains central to its design. Modern iterations incorporate critical safety enhancements and real-time monitoring capabilities, retaining the original vision: to improve gas exchange in patients with limited lung recruitability without increasing the risk of ventilator-induced lung injury (VILI).

This project is rooted in over 25 years of translational research, driven by clinician's determination to improve outcomes in critical respiratory failure.

1. **Preclinical Studies in a Porcine Model of Healthy and Injured Lungs**

Before initiating the first-in-human feasibility study, the VENTIJET system underwent an in-vivo validation protocol in a 39-kg male pig at the accredited experimental platform of IIS Biocruces-Bizkaia. The study followed institutional and national ethical approvals (OEBA-CET-2020-004; final authorization 2020/09), using standardized procedures for anesthesia, mechanical ventilation, invasive monitoring, lung injury induction, and structured ventilatory testing.

**2.1. Objectives**

The primary aim was to validate the safety and physiological behavior of the VENTIJET prototype across a wide range of respiratory mechanics—first in a healthy lung and then in an induced ARDS-like condition—before human application.

**2.2. Methods**

**Animal preparation and monitoring**

The pig underwent sedation, deep anesthesia, neuromuscular blockade, and intubation. A femoral arterial catheter, a jugular central venous catheter, a PiCCO system, a pneumotachograph and continuous capnography were placed, together with ECG, pulse oximetry and core temperature monitoring. This allowed real-time recording of flows, pressures, tidal volumes, esophageal pressure, hemodynamics, oxygen transport (DO₂/VO₂) and respiratory mechanics.

**Ventilatory protocol**

The study consisted of two major phases: a healthy-lung phase and an induced-injury (ARDS) phase.

**A) Healthy-lung phase**

The animal was initially ventilated using a conventional ventilator (Ohmeda 7800/Excel 210SE) until physiologic stabilization was achieved. It was then switched to the VENTIJET prototype, which was tested using two nozzle configurations: a standard nozzle and a high-performance nozzle. The two nozzles differed in their internal geometry: while the standard nozzle represents the baseline configuration, the high-performance nozzle incorporates a more tapered and narrower cone-shaped lumen, increasing jet acceleration at the outlet. This design results in higher effective delivered volume for the same inspiratory time and slightly higher PIP values, always within predefined safety limits.

Both configurations were evaluated across multiple ventilatory conditions, including tidal volumes of 6–8 mL/kg, respiratory rates of 20–30 bpm, FiO₂ of 0.4–0.56 and PEEP of 10 cm H₂O. Each setting was maintained for 15 minutes, with arterial and venous blood gases, ventilatory curves and hemodynamic variables collected at every step.

**B) Induced-lung-injury (ARDS) phase.**

Acute lung injury was induced using intratracheal hydrochloric acid (0.1–0.2 N, 5 mL/kg), producing a reproducible reduction in oxygenation and a 40–50% fall in compliance, sustained for at least 30 minutes. The same structured Ventijet protocol used in the healthy-lung phase was repeated under injured-lung conditions with both nozzles.

**2.3. Results**

**2.3.1. General safety and stability**

Across all experimental conditions—including variations in tidal volume, respiratory rate and nozzle configuration—the VENTIJET system maintained stable hemodynamics without episodes of hypotension, arrhythmias or cardiac output deterioration. Peak airway pressures remained controlled (typically 26–34 cm H₂O), end-expiratory pressure was predictable through the flow-dependent braking mechanism and no barotrauma (pneumothorax, pneumomediastinum or air leak) occurred. Temperature and humidification remained stable, and no device malfunctions were recorded.

These findings support a favorable safety profile under all tested conditions.

**2.3.2. Healthy lung physiology**

During healthy-lung ventilation with VENTIJET, SpO₂ remained 98–100%, PaO₂ ranged 160–214 mm Hg depending on FiO₂ and RR, and pH values were 7.45–7.58 with corresponding PaCO₂ values of 20–40 mm Hg. Compliance was preserved, and delivered tidal volume showed a linear relationship with programmed flow and inspiratory time. Hemodynamic variables (MAP, CO, SVR, DO₂ and VO₂) remained within expected physiological ranges.
Overall, the system provided predictable ventilation without evidence of hyperinflation or hemodynamic compromise.

**2.3.3. Injured-lung physiology (ARDS model)**

After acid-induced injury, compliance fell to 19–25 mL/cm H₂O and oxygenation and PaCO₂ changed as expected for ARDS physiology. Under these conditions, VENTIJET maintained adequate oxygenation (PaO₂ 119–245 mm Hg depending on FiO₂ and settings), preserved end-expiratory stability, facilitated ventilation even with low tidal volumes (6 mL/kg) at high respiratory rates (30 bpm) and did not induce hemodynamic instability.
These findings confirm the feasibility of continuous-flow ventilation under severely reduced compliance.

**2.4. Preclinical Safety and Translational Implications**

Under controlled experimental conditions, the VENTIJET prototype demonstrated a favorable safety profile and stable physiological performance in both healthy and injured porcine lungs. No barotrauma, hemodynamic deterioration or device failures were observed across a wide range of ventilatory settings.

Delivered tidal volume scaled predictably with programmed flow and inspiratory time, confirming the expected mechanical behavior of continuous-flow ventilation. Importantly, adequate gas exchange and end-expiratory stabilization were maintained even under ARDS-like conditions with markedly reduced compliance, without the need for excessive airway pressures. Comparable physiological responses were observed with both nozzle configurations, supporting reproducibility of the system’s performance.

Taken together, these findings provided the physiological and safety rationale to support the ethical and scientific progression to a first-in-human safety and feasibility trial.

1. **Technical Specifications and Operating Principles of the VENTIJET System**

**Pneumatic System**

VENTIJET operates on the principle of continuous flow jet ventilation, utilizing a custom-designed module known as the ECAF (*Equipo de Control y Acondicionamiento de Flujo*; *Flow Conditioning and Control Unit*). This unit contains the internal nozzle, responsible for accelerating gas flow to generate a high-velocity stream that functions as an expiratory brake. This mechanism increases end-expiratory pressure, promoting alveolar recruitment and reducing cyclic collapse.

Medical air or oxygen-enriched gas mixtures are delivered to the system through a controlled inlet and directed through the nozzle assembly. The pneumatic architecture allows precise adjustment of inlet gas flow—typically within the range of 30 to 60 L/min—as well as regulation of the inspired oxygen fraction through an integrated air–oxygen mixer. Inspiratory volume is manually controlled, accounting for circuit compliance, while inspiratory time and respiratory frequency are adjusted to define the ventilatory cycle. The ECAF interfaces directly with the patient’s endotracheal tube, minimizing circuit length and resistance and optimizing flow efficiency.

Supplementary **Figure 3** presents a schematic connection diagram of the VENTIJET prototype, outlining integration of the gas source, ECAF unit, control system, and patient interface.

**Electronic Control and Monitoring System**

The VENTIJET system incorporates an electronic control board that governs ventilatory timing cycles (inspiration and expiration), safety cutoffs, and visual and audible alarms. A touchscreen user interface enables real-time adjustment of ventilatory parameters, including respiratory rate, gas flow, inspired oxygen fraction, inspiratory pressure, and predefined pressure limits.

Pressure sensors and flow transducers embedded within the ECAF continuously monitor system performance, with all signals relayed to the control interface for immediate display and data export. Supplementary **Figure 4** shows the external view of the control unit, highlighting its compact casing, labeled connection ports, and touchscreen interface, while Supplementary **Figure 5** depicts the internal configuration, including the solenoid valve assembly, control wiring, and main electronic board components.

1. **VENTIJET continuous-flow architecture and inspiratory hold maneuver**
2. Functional valve logic:

The VENTIJET system operates on a continuous-flow architecture controlled by a three-solenoid valve state machine (S1–S3), which dynamically routes a constant upstream gas supply through the patient delivery pathway, the expiratory branch (toward the ECAF), or a dedicated bleed/relief pathway. This configuration allows precise control of gas routing across the different respiratory phases while maintaining continuous inlet flow.

**Table S1** summarizes the functional state of each solenoid valve and the resulting circuit-level behavior during inspiration, expiration, and static hold maneuvers.

**Table S1**: Functional valve logic across respiratory phases (engineering view).

| **Solenoid** | **Physical function** | **Pathway controlled** | **Inspiration** | **Expiration** | **Inspiratory hold** | **Circuit/physiological effect** |
| --- | --- | --- | --- | --- | --- | --- |
| S1 | Relief/bleed valve (controlled vent) | Vent to atmosphere or alternative discharge path for the continuous flow | Closed | Closed | Open | Provides an outlet for continuous flow when the patient circuit is occluded, preventing pressure build-up (critical during holds). |
| S2 | Patient delivery valve | Main flow path to the patient | Open | Open | Closed | Enables flow delivery during inspiration and maintains flow during expiration, introducing controlled resistance (expiratory braking). |
| S3 | Expiratory branch valve (patient → ECAF/outlet) | Exhalation pathway from patient to the expiratory module (ECAF/outlet) | Closed | Open | Closed | Allows exhalation during expiration; closes during inspiration and holds to block outflow and stabilize lung pressure/volume during the maneuver. |

1. Engineering rationale: continuous-flow holds and overpressure control

In a continuous-flow ventilation architecture, the upstream gas source maintains a non-zero inlet flow independently of the instantaneous state of the patient circuit. During inspiratory hold maneuvers, the clinical objective is to transiently isolate the patient interface to obtain static conditions, such as plateau pressure at end-inspiration.

However, in a continuous-flow system, simultaneous closure of both the patient delivery path and the expiratory branch without an alternative outlet would result in rapid pressure accumulation within the circuit. VENTIJET addresses this inherent risk by incorporating a dedicated bleed pathway controlled by solenoid S1.

During inspiratory hold maneuvers, solenoids S2 (patient delivery) and S3 (expiratory branch) are commanded closed to establish patient occlusion, while solenoid S1 is commanded open to divert the continuous inlet flow toward a controlled discharge path. This configuration effectively decouples two otherwise conflicting requirements: (i) achieving patient-side flow interruption to allow quasi-static measurements, and (ii) safely dissipating the continuous inlet flow to prevent overpressure within the ventilator circuit.

1. Control logic and synchronization of inspiratory hold maneuver

From a control-system perspective, inspiratory holds are implemented as clinician-initiated terminal substates within the respiratory state machine. When an inspiratory hold command is issued by the operator, the controller does not interrupt the ongoing respiratory phase. Instead, the maneuver is armed and automatically synchronized to the next appropriate phase boundary, namely the end of inspiration.

If the request occurs too close to the end-inspiratory boundary to be executed safely—due to timing constraints, valve actuation latency, or internal safety checks—the controller defers execution and applies the inspiratory hold at the end of inspiration of the subsequent respiratory cycle. Once initiated, the inspiratory hold is maintained until explicit clinician release.

A defining characteristic of the VENTIJET system is that, during the inspiratory hold substate, the continuous-flow source is explicitly routed through a dedicated bleed path (S1), rather than relying on uncontrolled leakage to manage pressure. This design decouples patient circuit occlusion from flow dissipation, allowing the patient interface to be transiently isolated while safely diverting the continuous inlet flow. As a result, stable static conditions are preserved at the patient interface, enabling accurate static pressure measurements while ensuring effective overpressure protection within the ventilator.

1. **Intellectual Property and Patent Registration**

The VENTIJET ventilator is protected under Spanish Patent No. ES2926416, granted by the Spanish Patent and Trademark Office (OEPM). The patent covers the design and operation of a continuous-flow ventilation system incorporating a nozzle-based expiratory brake, integrated safety features, and a real-time monitoring interface for ventilatory parameters. At the time of the study, the device had not been commercialized, and no financial revenues had been generated from this intellectual property.

**5. Phase-Based Analysis of Gas Exchange and Respiratory Mechanics in the human Safety and Feasibility Trial**

To explore the temporal evolution of respiratory mechanics and gas exchange during VENTIJET ventilation, a phase-based analysis was conducted across nine predefined time points. These included baseline and early phases under conventional mechanical ventilation with Puritan Bennett™ 840 (PB840), multiple time points during VENTIJET ventilation (VJ), and post-intervention phases following reconnection to conventional ventilation. Specifically, measurements were obtained at baseline and after 1 hour of conventional ventilation, during VENTIJET at 1, 6, 12, and 24 hours, and after reconnection to conventional ventilation at 1, 12, and 24 hours. While the main manuscript reports peak PaO₂/FiO₂ and respiratory system compliance (Crs) values, this detailed analysis illustrates the physiological progression across all study phases.

**Oxygenation**

The PaO₂/FiO₂ ratio showed a progressive increase during VENTIJET ventilation, reaching its highest values at 24 hours. In exploratory pairwise analyses, PaO₂/FiO₂ at VENTIJET 24 hours differed significantly from values observed at PB840 1 hour and during post-intervention PB phases (p = 0.025–0.042). The complete temporal profile of oxygenation across all phases is presented in Supplementary **Figure 7**.

**Respiratory System Compliance (Crs)**

Respiratory system compliance increased steadily over the course of VENTIJET ventilation. Exploratory comparisons demonstrated significant differences between Crs at VENTIJET 24 hours and several control phases under conventional ventilation (p = 0.028–0.039). The full time-course evolution of Crs is shown in Supplementary **Figure 8**.

**Lung Compliance (CL)**

Lung-specific compliance (CL) followed a similar upward trend during VENTIJET ventilation. Exploratory pairwise analyses identified significant improvements in CL over time, with p-values ranging from 0.011 to 0.031. Detailed phase-by-phase data are provided in Supplementary **Figure 9**.

**Driving Pressures (ΔPaw and ΔPL)**

Both airway (ΔPaw) and transpulmonary driving pressures (ΔPL) decreased progressively during VENTIJET ventilation. At 24 hours, exploratory analyses showed significantly lower ΔPaw and ΔPL values compared with baseline and post-intervention phases under conventional ventilation (p = 0.024–0.043). These findings suggest a more favorable pressure profile during VENTIJET ventilation, potentially reflecting reduced dynamic strain. Visual representations of these parameters are not included, as they were not part of the prespecified primary hypotheses.

**Expiratory Transpulmonary Pressure**

Expiratory transpulmonary pressure (PLexp) demonstrated a gradual reduction over time during VENTIJET ventilation, with significant differences observed in exploratory analyses (p = 0.015–0.039). This behavior is consistent with smoother expiratory transitions associated with the nozzle-based continuous-flow mechanism. The temporal evolution of PLexp is illustrated in Supplementary **Figure 10**.

Overall, this phase-based analysis indicates that VENTIJET ventilation was associated with adequate and sustained gas exchange alongside progressive and favorable changes in respiratory mechanics throughout the intervention period. These exploratory findings complement the primary analyses presented in the main manuscript and provide additional physiological insight into the behavior of continuous-flow ventilation in this first-in-human safety and feasibility study.

**6. Extended Safety Analysis: Adverse Events in the First-in-Human Safety and Feasibility Trial**

Adverse events (AEs) were prospectively recorded throughout the 24-hour VENTIJET intervention and during the immediate post-intervention period. No device-related serious adverse events (SAEs) occurred. All events were adjudicated by the clinical team based on temporal relationship, physiological plausibility, and compatibility with routine ICU care.

**Observed adverse events**

Three non-serious adverse events were recorded during the study, each occurring in a single patient.

The first event occurred in patient ID#04, who developed a brief desaturation to 87% during routine closed-circuit suctioning. This episode was attributed to the transient interruption of expiratory stabilization that occurs when jet flow is momentarily disrupted during suctioning. Oxygen saturation returned to baseline within seconds without requiring adjustments to VENTIJET settings. The event was mild in severity and considered unrelated to device performance.

The second event was observed in patient ID#08, who experienced a progressive decline in oxygenation despite stable hemodynamics and airway pressures. This patient had a history of heterogeneous, poorly recruitable ARDS and had required several previous prone cycles. Hypoxemia improved rapidly after re-proning, and the VENTIJET intervention continued uneventfully. This event was non-serious, mild-to-moderate in severity, and attributable to the patient’s underlying lung pathology rather than the ventilator.

The third event occurred in patient ID#10, who demonstrated sudden derecruitment with a drop in tidal volume and oxygen saturation following an accidental brief circuit disconnection during nursing care. The disconnection was promptly detected by the system, and the corresponding alarm was triggered as designed. Lung volume and gas exchange recovered fully after a short increase in FiO₂ and PEEP for two minutes. This incident was mild in severity and considered unrelated to the device, as similar derecruitment may occur with any ventilator following circuit disconnection.

**Events not observed**

No complications typically associated with invasive mechanical ventilation or continuous-flow systems were observed during the intervention or post-intervention period. In particular, no patient developed pneumothorax, pneumomediastinum, subcutaneous emphysema, hemodynamic instability, arrhythmias related to pressure load, respiratory acidosis (pH < 7.20), auto-PEEP accumulation, circuit obstruction, overpressure valve activation, humidification failure, unexpected pressure spikes, or device malfunction. This absence of complications supports a favorable safety profile for the prototype.

**Interpretation**

The adverse events observed were non-serious, transient, and fully explained by routine ICU procedures or underlying ARDS physiology. None required discontinuation of VENTIJET or modification of safety limits, and no event was judged to be device-related. No barotrauma, hemodynamic compromise, or device malfunction was detected. Overall, these findings indicate that continuous-flow ventilation with VENTIJET was well tolerated and feasible in a controlled ICU environment.

**REFERENCES**

Reference numbers correspond to those cited in the main manuscript.

**SUPPLEMENTARY FIGURES**

**
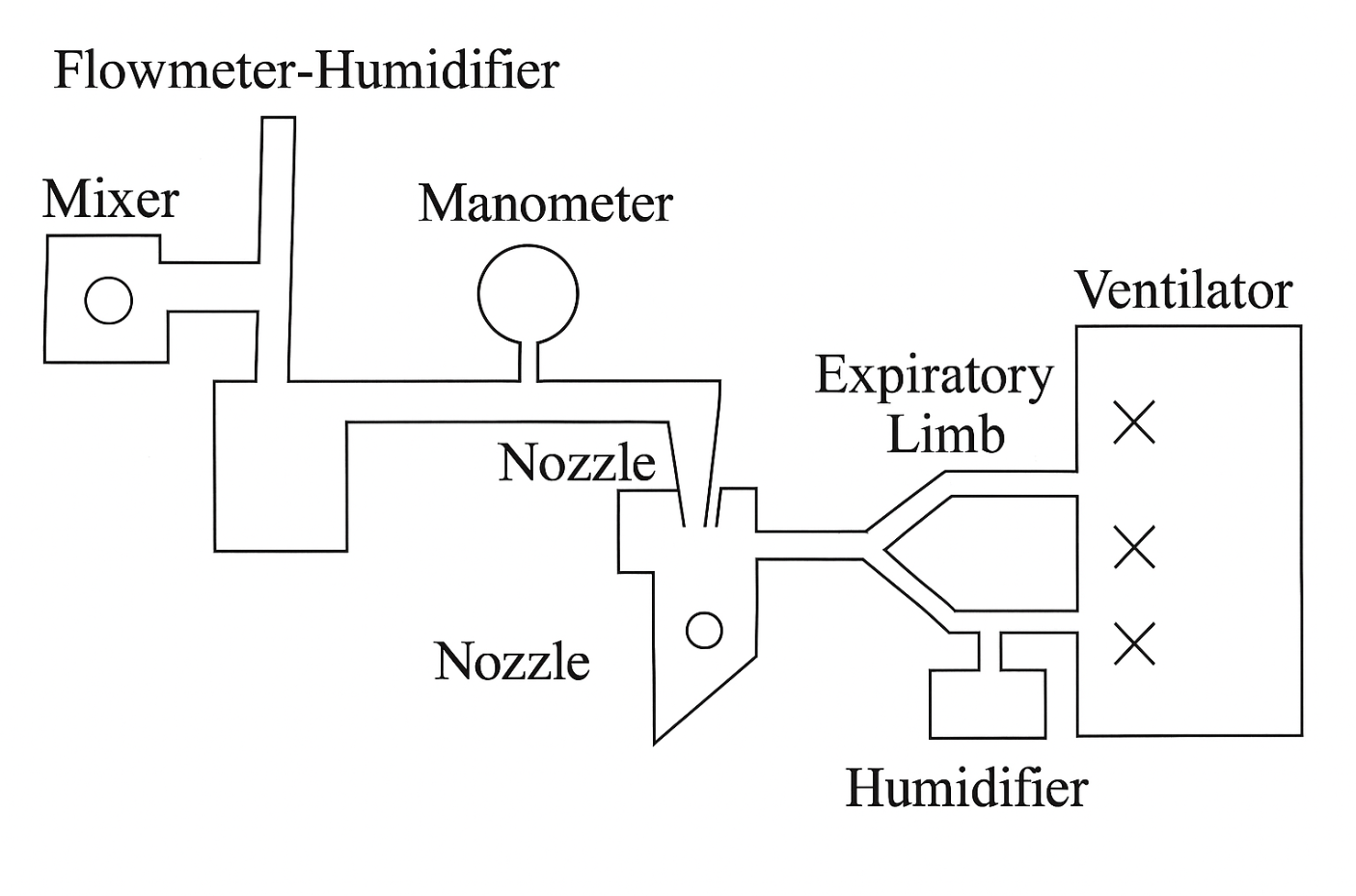
Supplementary Figure 1. Schematic diagram from Dr. Picazo’s original doctoral thesis.**Illustration showing the conceptual layout of the early VC-ET (Ventilación Continua Extratraqueal) prototype system, including jet flow generator, ventilator, and patient interface.

**
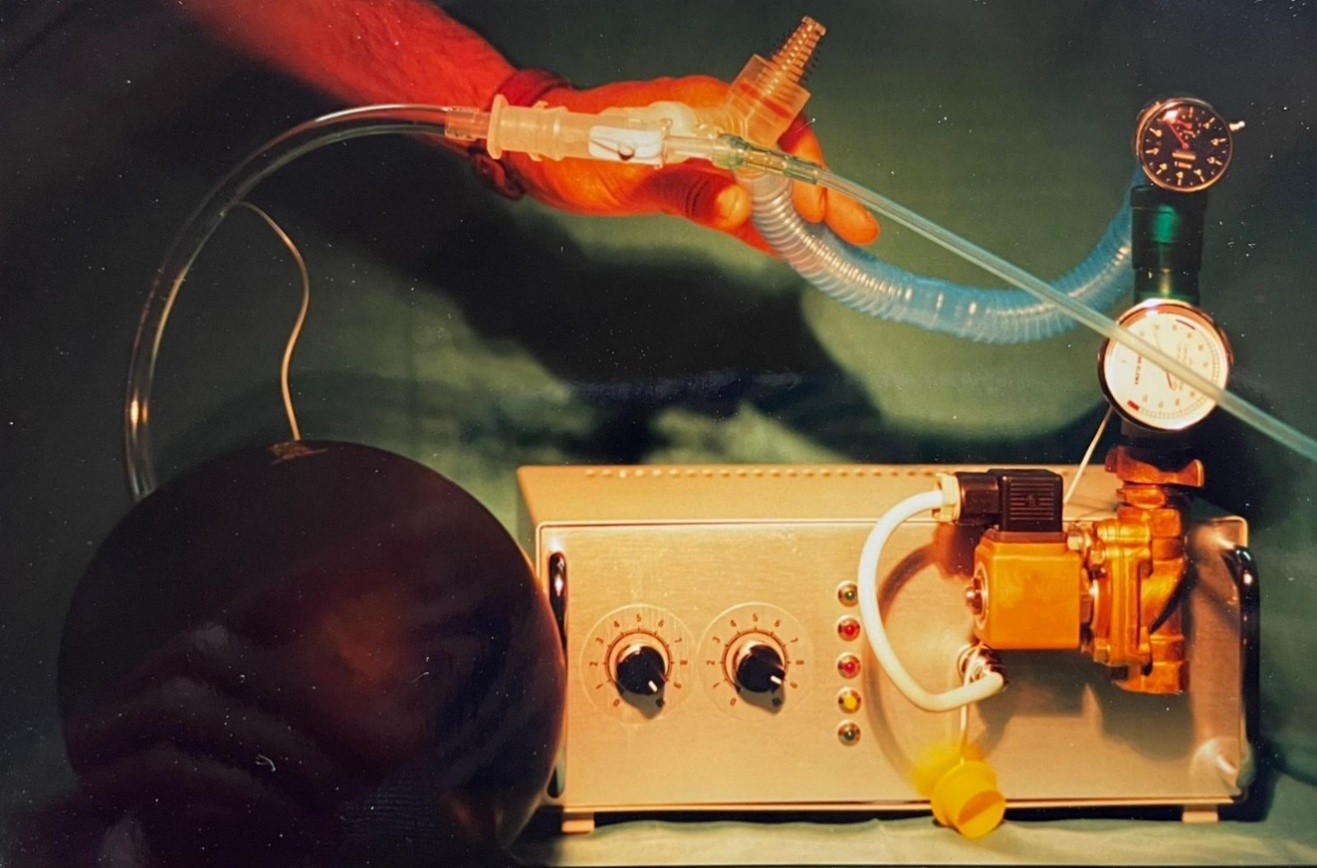
**

**Supplementary Figure 2. Original prototype of the VCJET ventilator developed by Dr. Lucas Picazo in the 1990s.** The device included manually adjustable flow regulators, a jet nozzle system, integrated manometers for real-time pressure monitoring, and a dedicated expiratory pathway. This early prototype was used in both experimental and clinical settings to deliver continuous extratracheal jet ventilation in combination with pressure-controlled ventilation, forming the conceptual foundation of the current VENTIJET system.


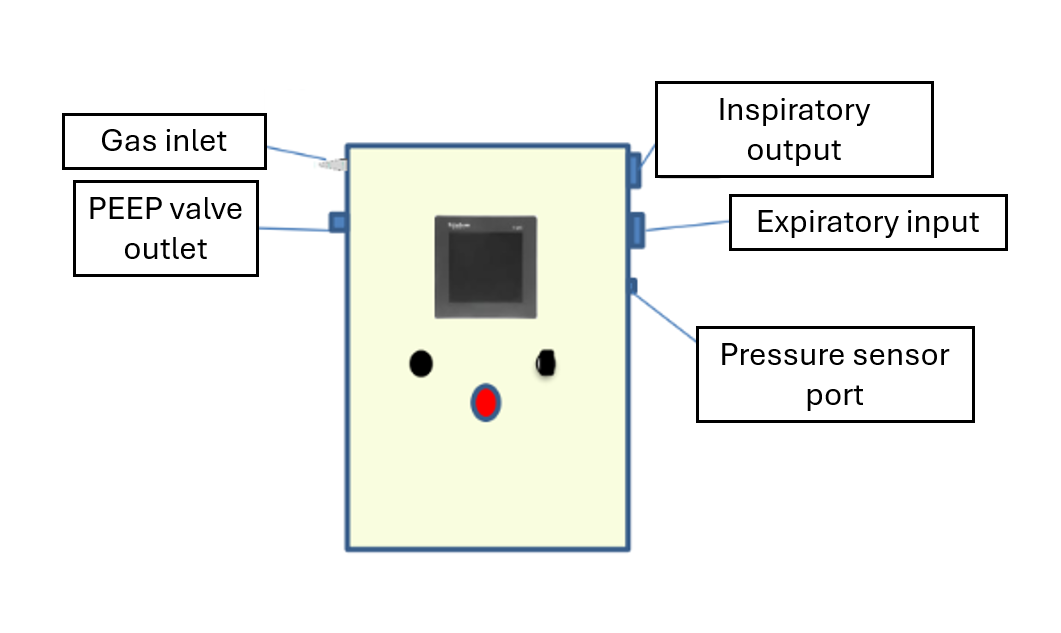
**Supplementary Figure 3. Connection diagram of the VENTIJET prototype.** Diagram representing the connection setup between the core components of the VENTIJET system: gas source, ECAF unit, control panel, and patient interface. This figure highlights the overall configuration and flow pathways of the current prototype.

**Supplementary Figure 4. External view of the VENTIJET ventilator.** Photograph of the VENTIJET system showing its compact structure, external casing, gas and power ports, and control panel interface, designed for use in critical care settings.


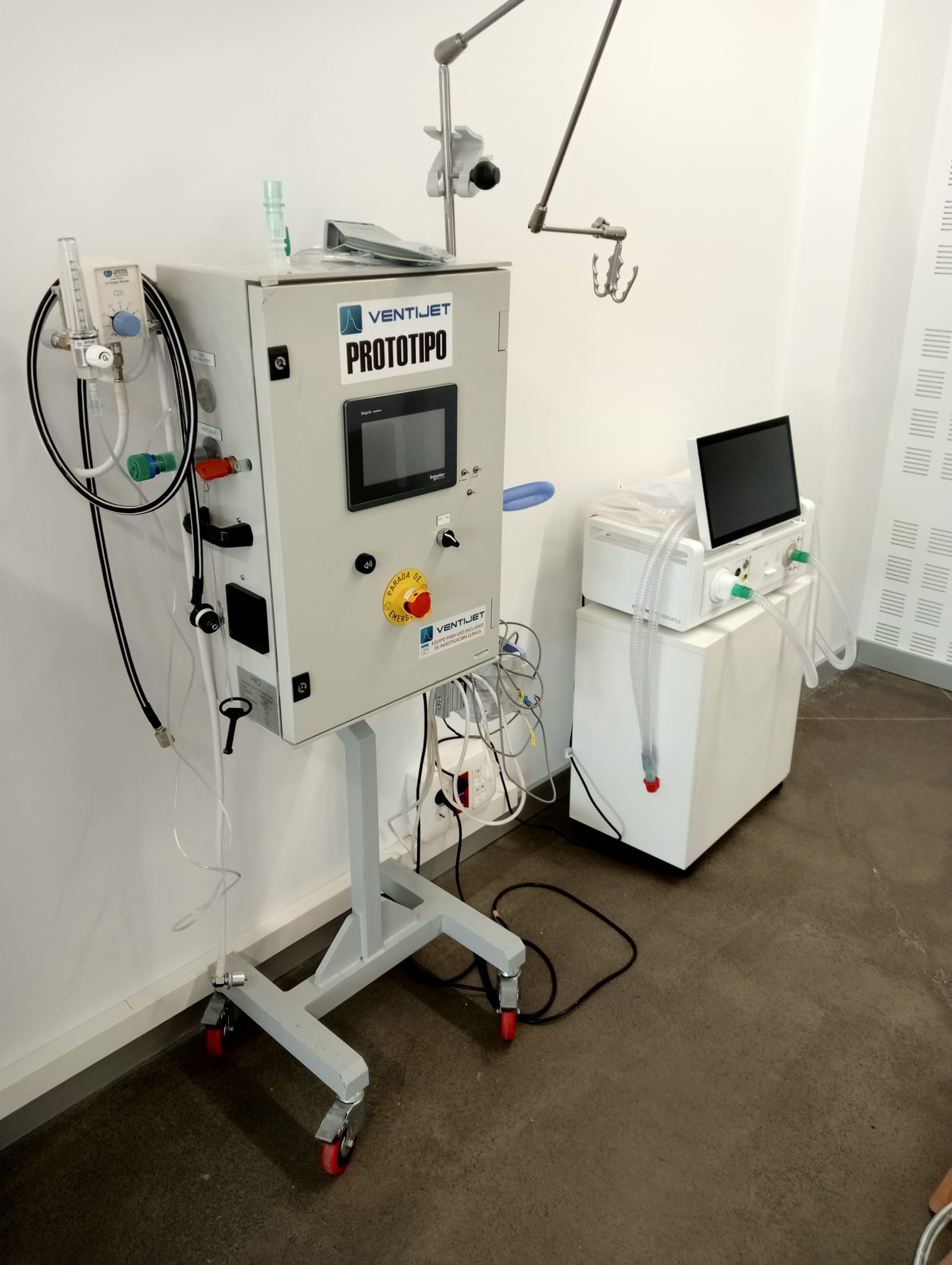


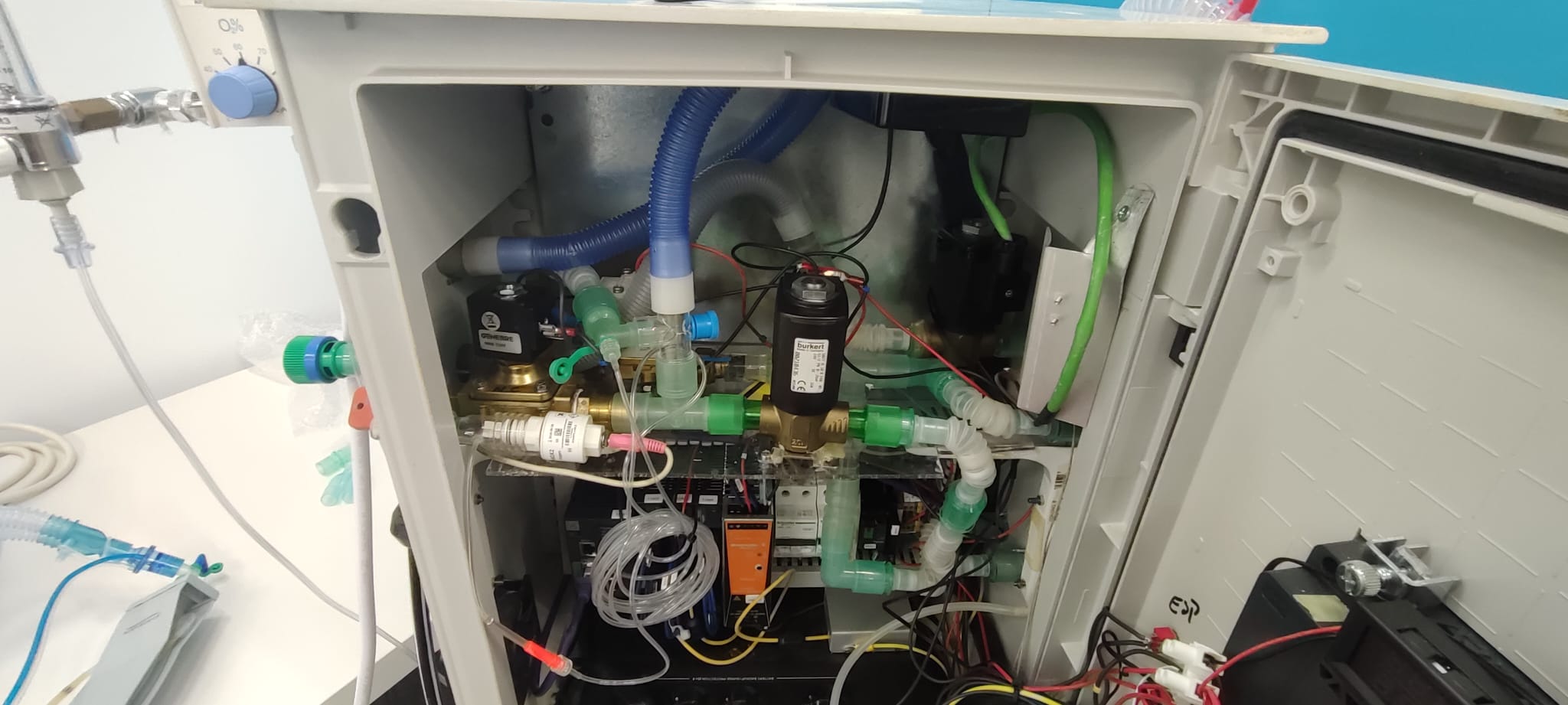


**Supplementary Figure 5. Internal view of the VENTIJET ventilator.** Photograph showing the internal components of the VENTIJET system, including the solenoid valve assembly, electrical wiring, and control board responsible for timing cycles and safety functions.

**
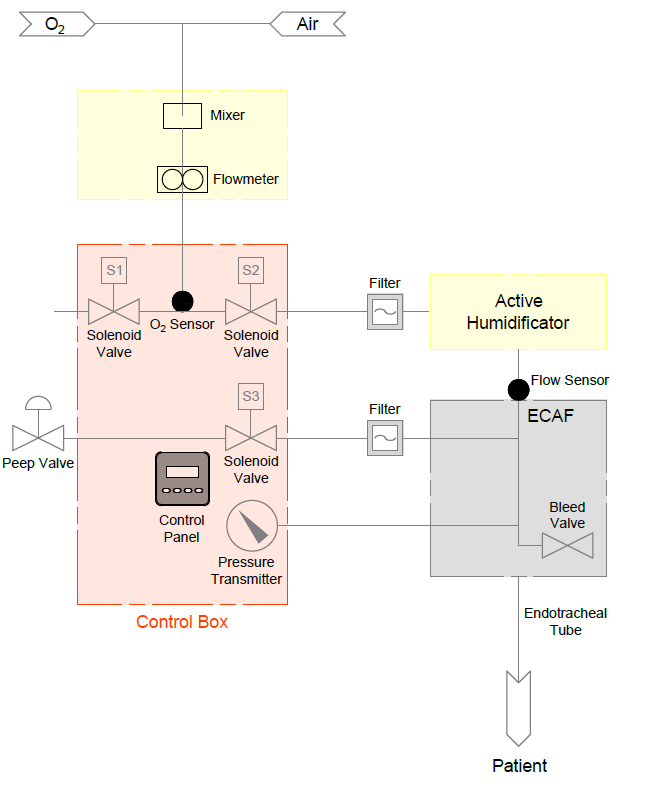
**

**Supplementary Figure 6: Functional schematic of the VENTIJET system illustrating valve configuration and flow routing during inspiratory hold maneuvers.** The diagram shows the continuous-flow architecture of the VENTIJET ventilator, including the main gas supply, control unit, solenoid valves (S1–S3), active humidification system, expiratory flow-braking module (ECAF), and patient interface.

**
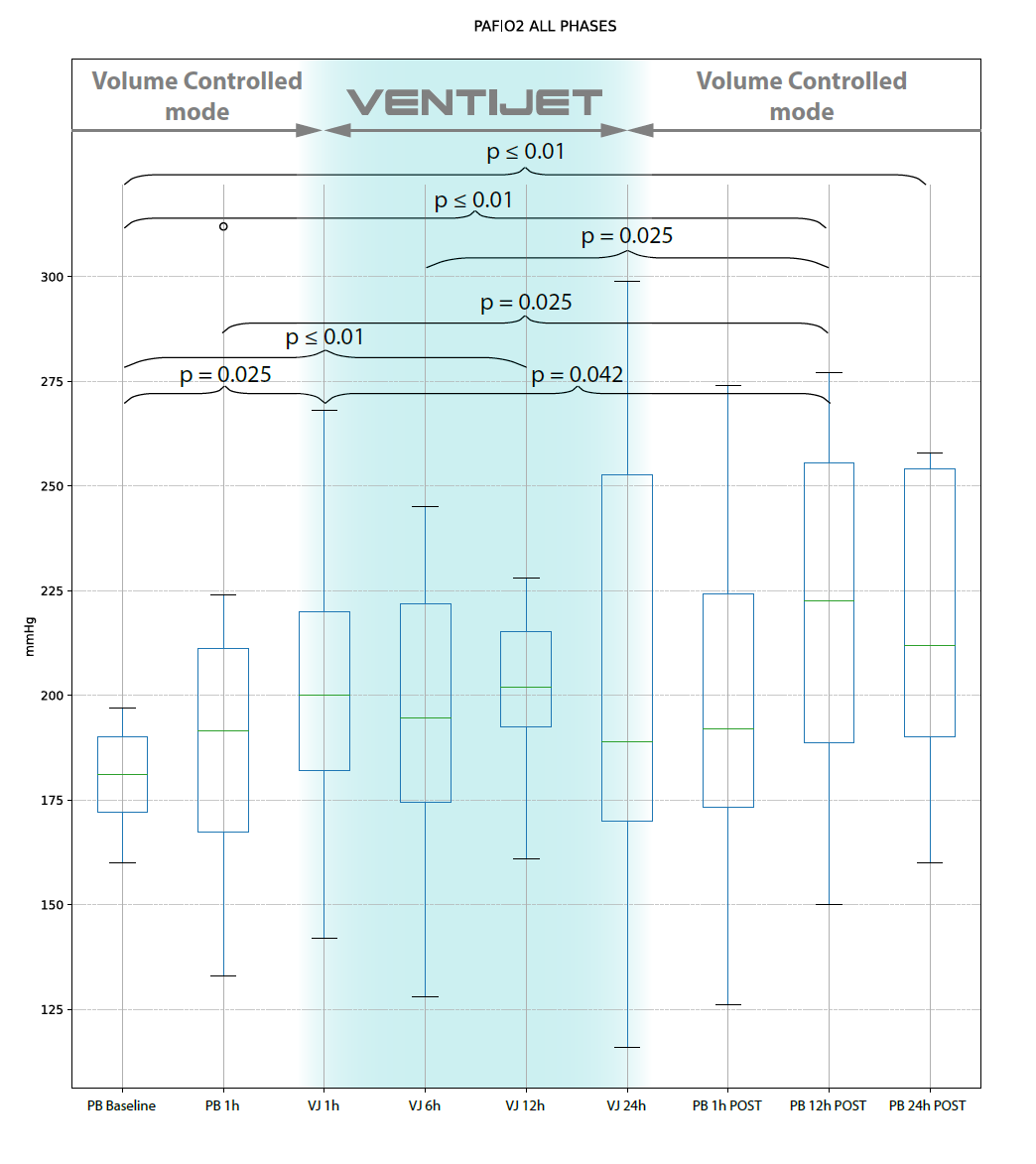
**

**Supplementary Figure 7. Time course of PaO₂/FiO₂ ratio across all study phases.** Boxplot showing PaO₂/FiO₂ evolution during baseline (PB Baseline), conventional ventilation (PB 1h), VENTIJET ventilation (VJ 1h, 6h, 12h, 24h), and post- VENTIJET conventional ventilation (PB 1h POST, 12h POST, 24h POST). Statistically significant pairwise comparisons are indicated.

**
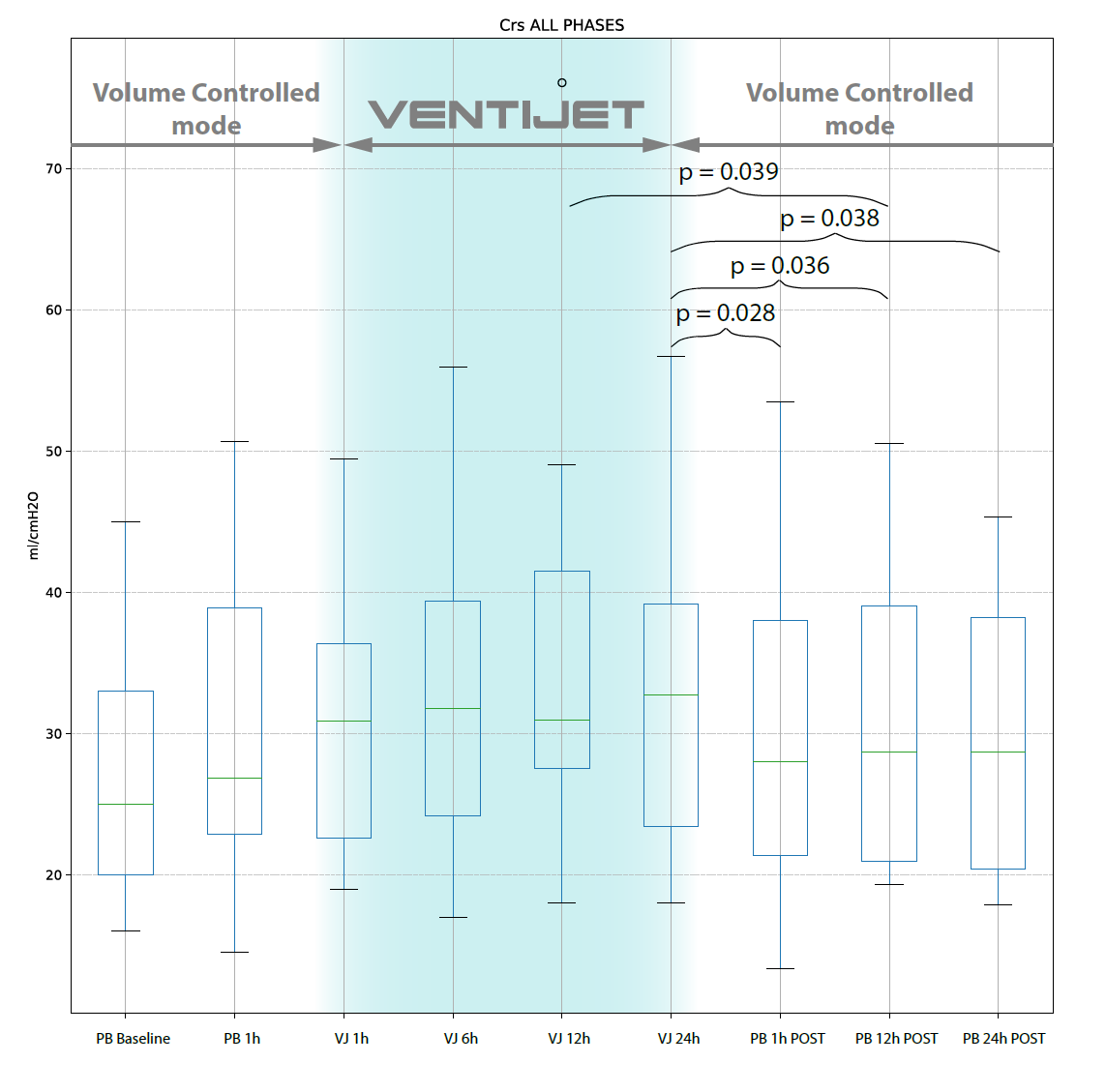
**

**Supplementary Figure 8. Time course of respiratory system compliance (Crs) across all study phases.** Boxplot showing Crs values during baseline (PB Baseline), conventional ventilation (PB 1h), VENTIJET ventilation (VJ 1h, 6h, 12h, 24h), and post- VENTIJET conventional ventilation (PB 1h POST, 12h POST, 24h POST). Statistically significant pairwise comparisons are indicated.

**
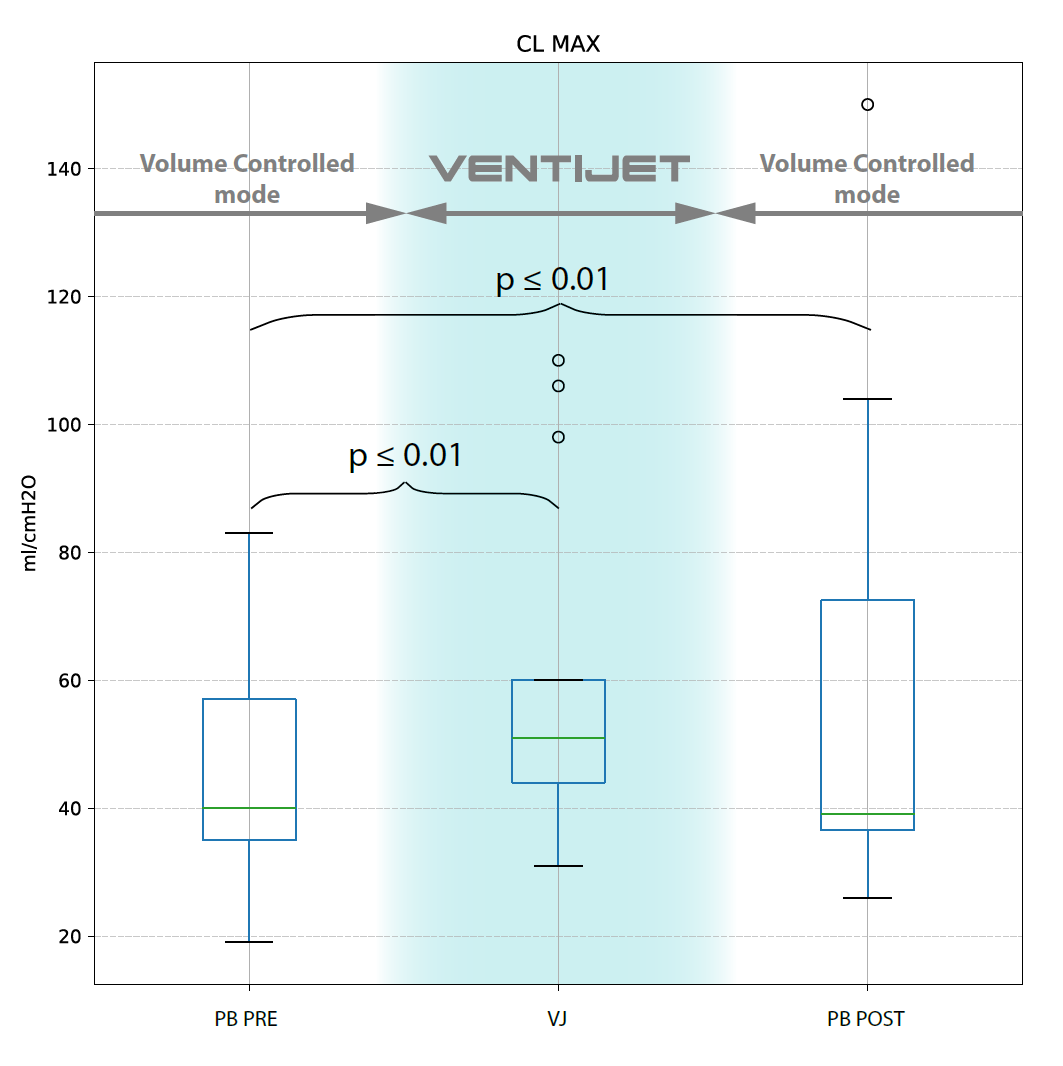
**

**Supplementary Figure 9. Maximum CL values across study phases.** Boxplots showing the highest observed values of lung compliance (CL) during each study phase: PB pre, VENTIJET (VJ), and PB post.


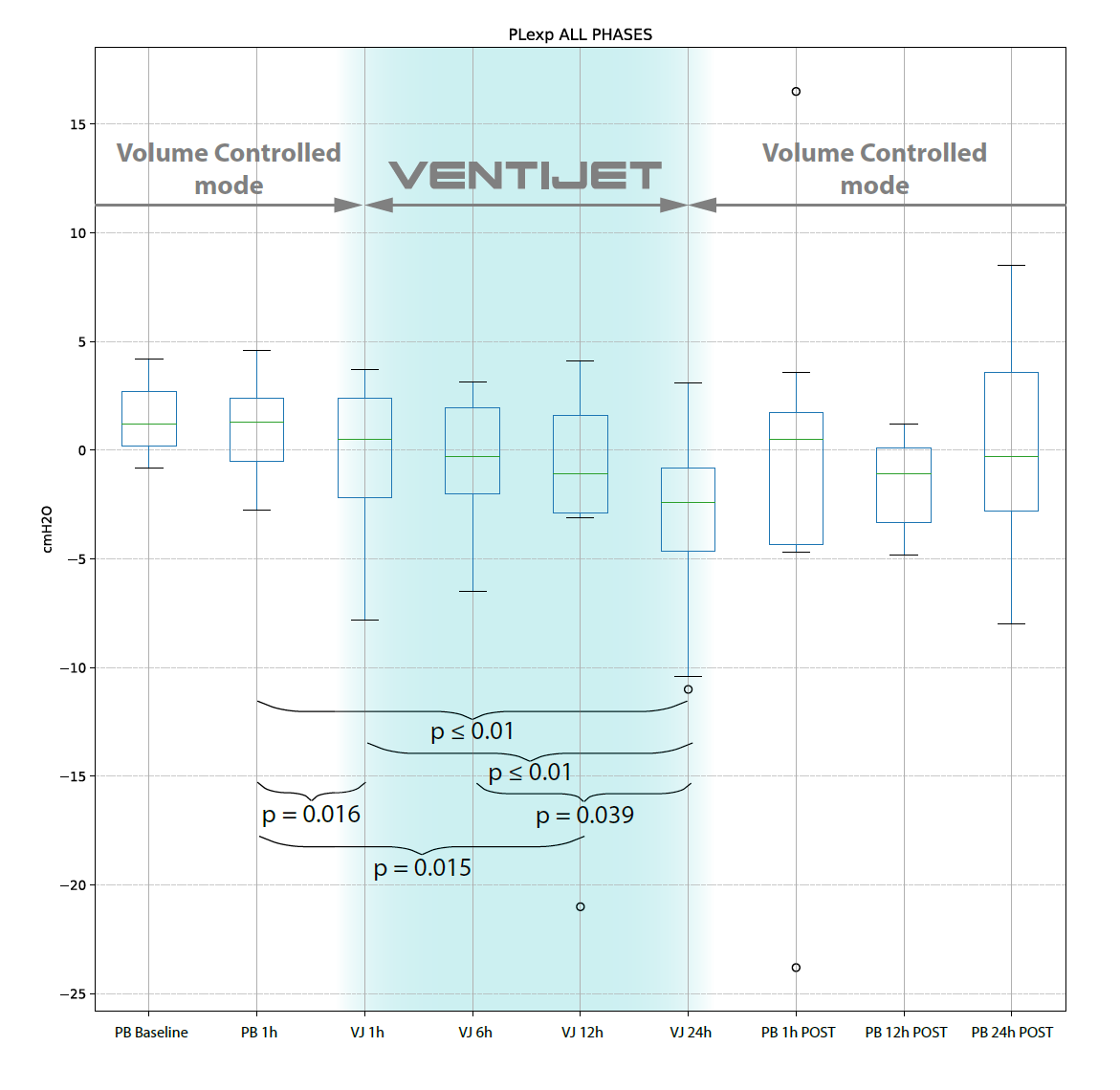


**Supplementary Figure 10. Expiratory transpulmonary pressure (PLexp) across study phases.** Median and interquartile ranges are shown. No significant changes were observed throughout the study period. PLexp values remained within physiological limits, suggesting stable end-expiratory transpulmonary pressure during VENTIJET use.
